# Addressing risks to biodiversity arising from a changing climate: The need for ecosystem restoration in the Tana River Basin, Kenya

**DOI:** 10.1371/journal.pone.0254879

**Published:** 2021-07-21

**Authors:** Rhosanna L. M. Jenkins, Rachel F. Warren, Jeff T. Price

**Affiliations:** 1 School of Environmental Sciences, University of East Anglia, Norwich, United Kingdom; 2 Tyndall Centre for Climate Change Research, University of East Anglia, Norwich, United Kingdom; Instituto Federal de Educacao Ciencia e Tecnologia Goiano - Campus Urutai, BRAZIL

## Abstract

Climate change is projected to have significant effects on the distribution of species globally, but research into the implications in parts of Africa has been limited. Using species distribution modelling, this study models climate change-related risks to the terrestrial biodiversity (birds, mammals, reptiles, amphibians and plants) of Kenya’s economically-important and ecologically diverse Tana River Basin. Large reductions in species richness are projected with just 2°C warming (relative to preindustrial levels) with birds and plants seeing the greatest impact. Potential climate refugia for biodiversity are identified within the basin, but often overlap with areas already converted to agriculture or set aside for agricultural expansion, and the majority are outside protected areas. Similarly, some protected areas contain no projected refugia at higher levels of global warming, showing they may be insufficient to protect the basin’s biodiversity as climate changes. However, risks to biodiversity are much smaller if the Paris Agreement’s goal of limiting global warming to ‘well below 2°C’ warming, rather than 2°C only, is met. The potential for refugia for plants and animals decreases strongly with warming. For example, 82% of the basin remaining climatically suitable for at least 75% of the plants currently present at 1.5°C warming, as compared with 23% at 2°C and 3% at 4.5°C. This research provides the first assessment of the combined effects of development plans and climate change on biodiversity of the Tana River Basin, including identifying potential areas for restoration, and contributes to a greater understanding of biodiversity protection and adaptation options in Kenya.

## Introduction

Climate and land use change have been and will continue to be the two most significant threats to global biodiversity [1, 2]. The substantial risk that climate change poses to biodiversity at the global scale [3, 4] and species’ responses, in particular through shifts in ranges, are widely researched [5, 6]. Biodiversity has been responding and adapting to changes in climate throughout history, but many species may be ill-equipped to deal with the current, more rapid rate of warming [7]. There will be species that prefer the warmer climates and become more abundant [8] but, for many, higher temperatures will lead to an increased extinction risk or need to be able to move to cooler areas. Many studies have already observed shifts in species’ ranges and attributed this to climate change [9, 10]. Mobile species, such as butterflies, may be more able to track changes in climate; less mobile species, including many plants, tend to lag behind [11]. This means that groups of plants and animals have varying levels of vulnerability. Significant risks to global biodiversity are likely even if global temperature rise is limited [12, 13] and the Paris Agreement goals of constraining warming to well below 2°C are met, so understanding which areas and species are most at risk is vital.

Research into the impacts of climate change on biodiversity rarely also considers the effects of projected future land use change which will occur alongside changes in global temperatures and weather patterns [14]. Land use and cover is constantly changing across the world, as a result of multiple drivers and impacts, which can contribute to both climate change and biodiversity loss [15, 16]. The greatest global change in land use has been towards more agricultural land [17]. As settlements and agriculture expand, more land is converted from its natural state. In addition, road and rail networks dissect the landscape, splitting areas of similar vegetation into smaller fragments. Increasingly fragmented habitats will limit species’ abilities to respond to climate change through dispersal and tracking their preferred climates. Species that remain in isolated habitat fragments will begin to experience other negative effects, including potential reductions in natural genetic variation within the population and even local extinctions [18]. Preserving biodiversity is not only important in order to save species but also for wider ecosystems and human society. Reductions in biodiversity will lead to losses of ecosystem function and services, which will have impacts on humans [19–21].

Many published studies on the effects of climate change on terrestrial biodiversity in Africa have focused on southern Africa [22–25] or Madagascar [26, 27]. Research into the implications of climate change for terrestrial biodiversity of East African countries is more limited. Previous studies focusing on Kenya have largely only considered single species, such as Grevy’s Zebra [28] and Rothschild’s Giraffe [29]. The biodiversity of Kenya is already under threat from a myriad of sources, including ecosystem degradation, water scarcity and habitat fragmentation [30]. Most threats will be exacerbated by climate change and socio-economic development (through the changes in land and resource use). East Africa is also a region where the global climate models (GCMs) disagree on the possible changes to precipitation; with some GCMs projecting reductions in rainfall while many project increases [31, 32]. This uncertainty increases the difficulty in planning adaptation in this region. For example, there is a significant amount of work on how GCMs may misrepresent the rains in East Africa [33, 34].

Kenya’s Tana River Basin has previously attracted scientific interest, including studies on the impacts of dam construction [35], the ecological importance of the lower section of the basin [36] as well as more recent investigations into the impacts of climate change on the hydrology of the area [37, 38]. The Government of Kenya (GoK) has outlined significant development targets and infrastructure investments for hydropower, domestic water provision and irrigation in the region as part of their national development blueprint, the Vision 2030 [39]. The Tana River Basin already plays a vital role in the country’s economy; supplying 80% of Nairobi’s drinking water and around 70% of Kenya’s hydropower energy through its dams and is likely to become even more important if the Vision 2030 is realised. GoK’s Vision 2030 [37] also aims to ensure that ‘all wildlife ecosystems are fully protected’ and that wildlife corridors are secured. The basin is a biodiversity hotspot: it contains 15 Key Biodiversity Areas [40], its delta ecosystem has been designated as a Ramsar wetland and it is within two WWF Priority Places, the East African Acacia Savannas and the East African Coastal Forests [41]. In order to achieve the aim of protecting ecosystems, the combined effects of land use and climate change on biodiversity must be established.

Land degradation increases the vulnerability of biodiversity. 36% of the Tana River Basin has already been converted to agriculture, so some ecosystem restoration may be necessary to best protect species in a changing climate. The United Nations declared 2021–2030 as the UN Decade on Ecosystem Restoration, which aims to restore degraded land to help tackle the climate crisis and protect natural resources. This restoration goes beyond benefits to biodiversity, as it also increases societal benefits derived from ecosystem services especially carbon removal.

This study uses the outputs of species distribution models to assess the impacts of projected climate change on the biodiversity (mammals, birds, reptiles, amphibians and plants) of Kenya’s Tana River Basin. It will determine the presence of climate refugia and the extent to which they are threatened by agriculture and development activities, and identify areas where biodiversity would benefit most from focused restoration efforts in the context of a changing climate. This study is the first to use the recent Government of Kenya development plans and compare them to projected changes in species distributions in Kenya. By identifying climate refugia and using their locations to show the trade-offs between proposed development and biodiversity conservation, the results of this research will be useful to those seeking to protect the biodiversity of Kenya in a changing climate.

## Materials and methods

### Study area

**Fig 1:**
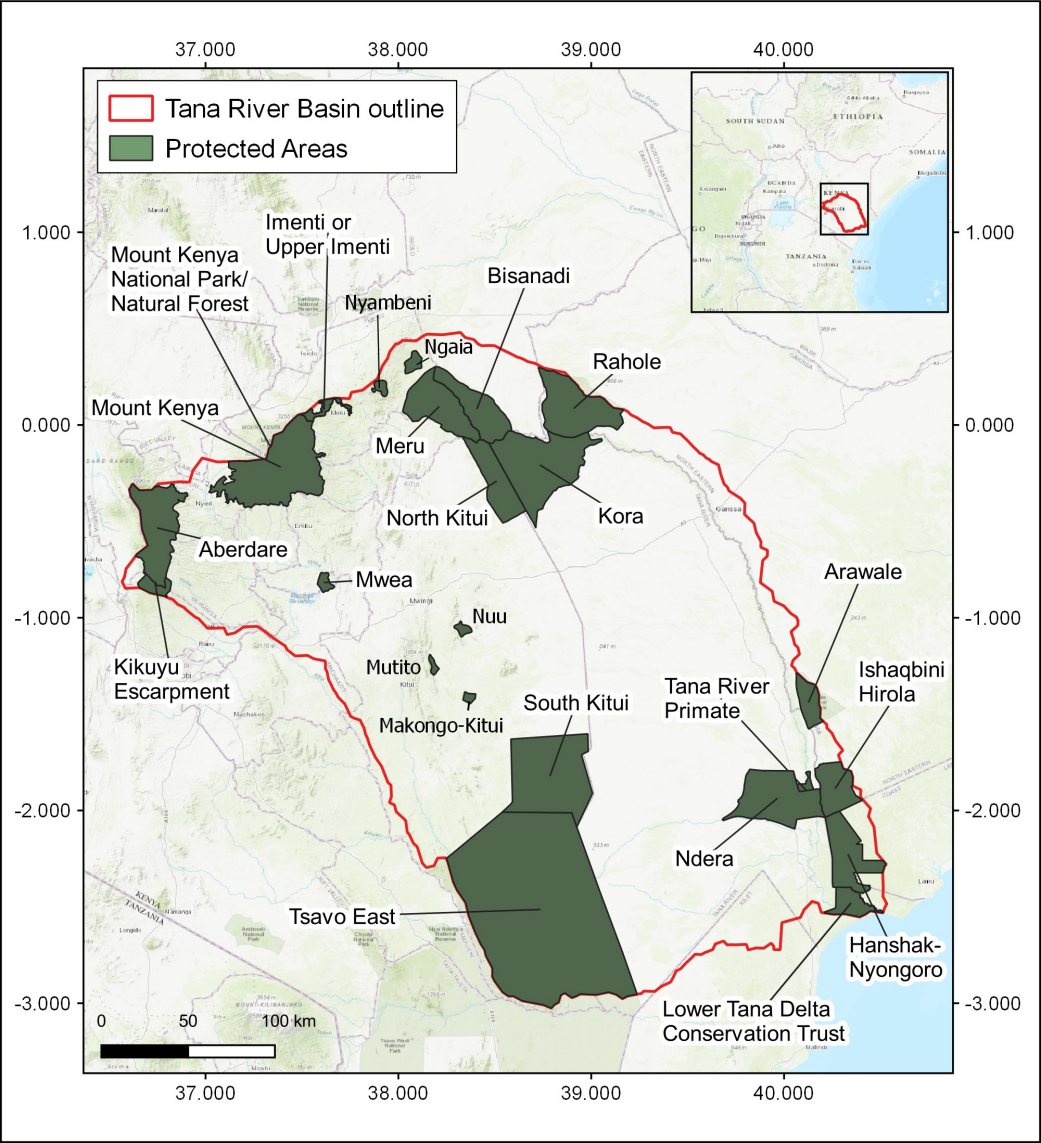
The Tana River Basin. This map shows the locations of protected areas considered in this study. Protected Areas data was obtained from the World Database of Protected Areas [44].

The Tana River Basin (Fig 1) is located in South-eastern Kenya and covers around 95,000km^2^; 20% of the country’s total land area. At approximately 1000 km from source to mouth, the Tana River is the longest river in the country, originating from the southern slopes of Mount Kenya and flowing into the Indian Ocean through the Tana Delta. The land type varies greatly within the basin, with the highest elevations classified as humid; central and coastal areas as semi-arid; and the remainder as arid.

The majority of the population of the Tana River Basin lives in rural areas. Populations in Kenya are concentrated around the wetter areas and high potential agricultural areas [42]. Population growth rates in the Tana Basin are relatively low compared with other catchments in the country [43]. However, even this population increase is likely to put increased pressure on limited water and land resources [35]. The population growth is particularly significant in the upper basin, where higher numbers of people are leading to land shortages and increased land degradation. Peoples’ livelihoods within the basin comprise a wide range of activities, including fishing, agriculture and pastoralism, as well as work related to conservation and employment within urban areas [43].

The basin contains 43 protected areas (PAs) either in whole or in part (Fig 1). Forest reserves of less than 20km^2^ and private ranches were not included in this research due to the spatial scale of the data used (20km^2^). S1 Table shows the full list of PAs considered here and their designation and size in km^2^ [44]. In the upper reaches of the Tana River, the slopes of Mount Kenya and the Aberdare range are protected as a National Park or as forest reserve. The north of the basin also includes PAs adjacent to the main Tana River, such as Meru and Kora National Parks (northeast). Much of the floodplain adjacent to the lower reaches of the river around the Tana Delta region is protected as community nature reserves and also contains the Tana River Primate Reserve. The Tsavo East National Park and South Kitui National Reserve are located in the southwest of the basin, furthest away from the Tana River itself. Many species, such the African lion and buffalo, are already largely confined to PAs so they provide important habitats for the region’s biodiversity. Endemic Bird Areas are located in the mountains in the Upper Tana and at the coast around the Tana Delta. The basin is home to several species of endangered primates, including the Tana River Red Colobus (*Procolobus rufomitratus*) and the Tana River Mangabey (*Cercocebus galeritus*), which are both endemic to the area [45, 46].

### Species distribution modelling and the Wallace Initiative

This study uses models developed by the Wallace Initiative, a global project designed to assess climate change impacts on the distribution of species [13, 47]. It links outputs from ClimGen [48] with the species distribution model, MaxEnt [49], and data from the Global Biodiversity Information Facility [50]. The current version of the Wallace Initiative has a spatial resolution of 20km x 20km and uses a range of 21 alternative climate models (GCMs) in order to account for uncertainties in the projections. For this research, five terrestrial taxa (plants, amphibians, birds, mammals and reptiles) were analysed.

The Wallace Initiative projects changes to species distribution with specific levels of global warming above pre-industrial levels: those corresponding to the Paris Agreement global temperature targets of 1.5 and 2°C; the nationally-determined contributions (NDCs) which have a lower estimate of 2.7°C and an upper estimate of 3.2°C; and a no-mitigation ‘business as usual’ scenario of 4.5°C [51].

This research involved three steps. First, the extent to which the terrestrial biodiversity of the Tana River Basin is affected by climate changes was determined. Specific changes considered are (i) proportion of species richness lost, and (ii) presence of climate refugia. Within the Wallace Initiative, refugia are defined as cells where, in at least 11 of the 21 GCMs used, at least 75% of the current species richness can remain in a changing climate. Current land use, and potential land use change are excluded from this mapping of refugia.

Secondly, the extent to which the projected climate refugia are threatened by present and potential future land use was assessed. The locations of projected refugia were compared to (i) locations of protected areas, (ii) current agriculture within the basin, to determine which potential refugia have already been converted to alternative land use, and (iii) spatial information from management plans produced by the GoK to determine the areas most at risk from future development. Data on the location of agricultural land was obtained from the Land Cover (LC) project of the European Space Agency (ESA) Climate Change Initiative (CCI), which is a global land cover map at 300m spatial resolution [52]. The agricultural land classes were extracted and reclassified. This is necessary in order to determine whether historical land use change has altered the landscape in a way potentially preventing the movement of species and restricting their ability to respond to climate change. These areas could also be important areas for the focus of restoration activities. Spatial information from the National Spatial Plan 2015–2045 (NSP; [53]) which was produced by the Government of Kenya, was digitised using GIS software as original digitised files were not readily available.

Third, the importance of facilitating movement was considered. The Wallace Initiative employs different dispersal scenarios to examine whether species can move to track their preferred climate. Within the Wallace Initiative, dispersal refers to the average long-term shift of an entire species’ range based on a review of rates in the published literature [13]. It is the geographic speed of the whole population rather than the velocity of movement of individuals across a species’ climatically suitable geographic range (climate envelope) which is being measured [10]. The previous steps of this research only considered a scenario in which species are not able to shift their geographic ranges. This is in common with many previous studies [54] and reflects the fact that, in practise, many species’ ability to shift their ranges will be severely limited by the present land use. However, it is likely that some species will be able to move with sufficient speed to shift their ranges in response to warming, so a sensitivity analysis was also carried out to show the potential importance of facilitating species’ movement.

Warren et al. [13] reviewed average dispersal speeds in order to create a scenario where range shifts may occur at a realistic rate. This review concluded that mammals and birds are able to disperse at a realistic rate of, on average, around 1.5 km/yr, while plants, amphibians and reptiles are only able to disperse at 0.1 km/yr on average. The inclusion of a realistic dispersal scenario is a key benefit of the Wallace Initiative and this can be seen as a proxy for climate-smart conservation management, where protected areas are linked by corridors and species can disperse. Full dispersal, which has frequently been previously used in species distribution modelling, has been deemed to be unrealistic, due to factors such as barriers to species movement, a lack of instantly available suitable habitats and the dynamics of range shifts that have previously been observed [13, 55]. For this research, the difference between projected changes in species richness when species are able to shift their range compared to when they are unable to move beyond their current geographic range has been examined for mammals and birds as a sensitivity analysis to examine the potential importance of facilitating movement. Based on the realistic dispersal rates described above, birds and mammals may be able to move rapidly enough to remain in equilibrium with the climate. Based on the low dispersal rates found in the published literature, it has been assumed that plants, amphibians and reptiles are unlikely to be able to move at a sufficient speed to track their preferred climate. Therefore, this analysis has not been included for these taxonomic groups.

## Results

### Proportions of species at risk from climate changes

**Fig 2:**
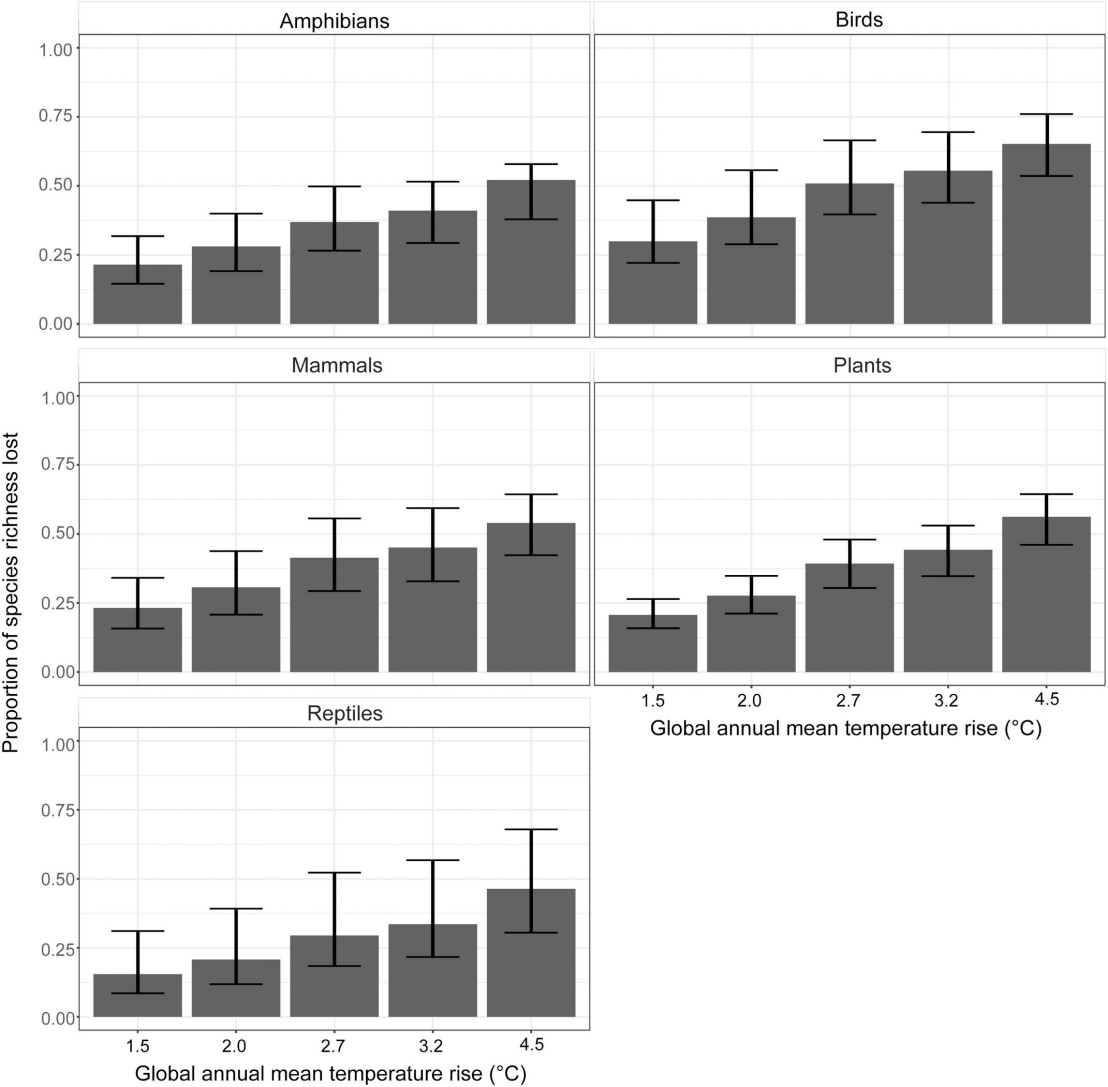
Basin-average proportion of species richness lost with different levels of global temperature rise. Data are presented as the mean across 21 GCMs, with error bars indicating the 10 to 90% range.

Results show that climate change poses a significant threat to the biodiversity of the basin. In most cases, a significant proportion of current modelled species richness could be lost (Fig 2). For all five taxa, the proportion at risk increases steadily with higher levels of warming. With 2°C warming, a basin-average of 38% of birds and 31% of mammals could see a loss of their suitable climates. With 4.5°C warming, 65% of birds and 54% of mammals could be lost from the basin. For plants, 56% of modelled species could be at risk with 4.5°C of warming. Similarly, around 50% of reptiles and amphibians could be lost from the area under the business as usual scenario.

### Climatic refugia

**Fig 3:**
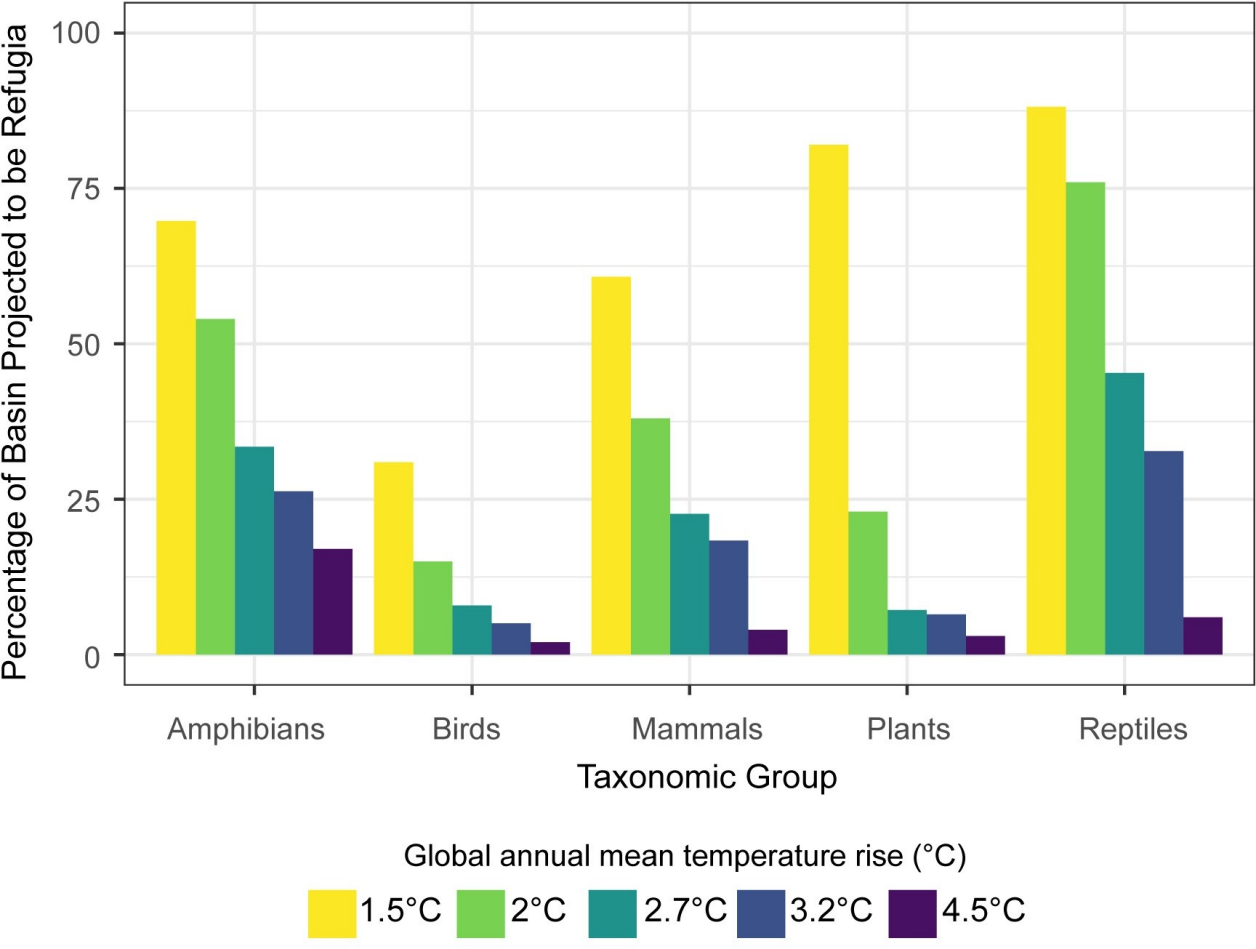
Percentage of the Tana River Basin projected to contain refugia with different warming levels. Refugia are areas where at least 75% of the current species richness is able to persist in a changing climate by 11+ GCMs for the five taxa (amphibians, birds, mammals, plants and reptiles) with different global temperature increases.

Climate refugia were identified for all five taxa within the Tana River Basin. When warming is limited to 1.5°C, the greatest proportion of the basin is projected to be refugia for all taxa, ranging from 88% of the basin for reptiles to 31% for birds (Fig 3). Limiting global temperature rise is shown to be particularly beneficial for plants, which means that animals may be benefitted indirectly as they depend on plants for survival (as they provide habitat and food sources). 82% of the basin is projected to be refugia for plants with 1.5°C but only 23% if warming reaches 2°C. This is reduced to 3% with 4.5°C warming.

**Fig 4:**
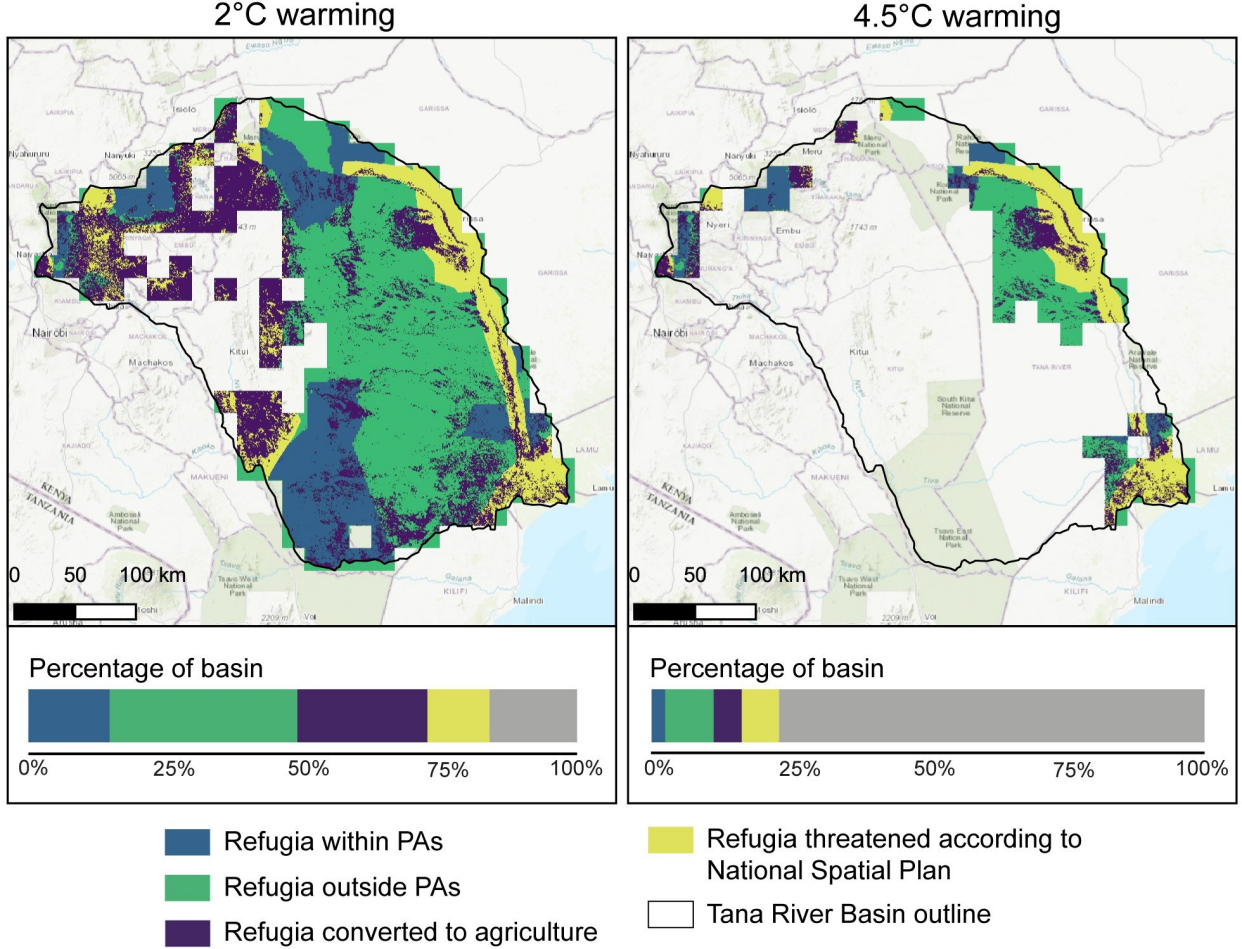
Locations of refugia with 2°C and 4.5°C warming. Refugia are projected only if 11 or more GCMs agree on their presence for at least one of the five taxa considered in this study. Bars show the percentage of the basin within each of the categories. Grey indicates the percentage of the basin which is not projected to contain refugia. Protected Areas data was obtained from the World Database of Protected Areas: [44].

When warming is limited to 2°C, around 81% of the basin (76,600 km^2^) is projected to contain refugia for at least one taxonomic group, including 18% within PAs, although some of this land has already been converted to agriculture (Fig 4). 76% of the basin is considered refugia for reptiles, 54% for amphibians, 38% for mammals, 23% for plants and 15% for birds. Only areas in the west of the basin are not projected to be refugia for amphibians (S1 Fig) and reptiles (S5 Fig). For birds, mammals and plants (S2–S4 Figs), refugia are concentrated around the mountains in the north, along the main river in the east of the basin and the Tana Delta in the southeast. The Hanshak-Nyongoro Community Conservancy, Ishaqbini Hirola Community Conservancy, Lower Tana Delta Conservation Trust and Ndera Community Conservancy in the south of the basin are projected to contain refugia by most models. Significantly, there are cells within the basin where all 21 GCMs project refugia for all four animal taxa with 2°C warming. These areas where the models are in agreement may prove important areas to focus conservation resources. Approximately 29% of the projected refugia at 2°C warming have already been converted to agriculture.

With warming of 4.5°C, only 21% of the basin is projected to contain refugia for at least one taxonomic group, including 11% within PAs (Fig 4). Of this, nearly a quarter (23% of projected refugia) has already been lost to agriculture. These ‘lost refugia’ often border PAs; particularly in the north of the basin and in the delta region in the southeast. For most taxonomic groups, refugia are concentrated within and around Mount Kenya and Aberdare National Parks in the north of the basin and around some PAs in the delta region. For amphibians, a large area in the east of the basin is also projected to be refugia with these high levels of global temperature rise (S1 Fig). Less than 1% of the basin is projected to act as refugia for all five taxa (S6 Fig).

Agriculture is present to some extent in all of the protected areas examined in this research. Over 20% of the land in Imenti or Upper Imenti Forest Reserve, the Tana River Primate Reserve, Kora National Park, Kikuyu Escarpment, Mwea National Reserve, South Kitui National Reserve, Ishaqbini-Hirola Community Conservancy, the Lower Tana Delta Conservation Trust and Hanshak-Nyongoro Community Conservancy is identified by remote sensing as agricultural land.

Proposed land use changes detailed in the GoK’s National Spatial Plan [52] (S8 Fig) will have implications for wildlife and plants, both in terms of current PA management and potential climate refugia. The area in the north of the basin contains most of the high and medium potential agricultural land, as well as large, proposed irrigation areas and a proposed economic growth area. Another area of proposed irrigation land runs next to the river in the lower part of the basin. In total, these activities cover over 36,000 km^2^ (38% of the basin). Proposed hydropower stations will be situated along the main river, further downstream than the existing hydropower stations. The proposed irrigation area in the upper basin appears to coincide with these hydropower stations and dams. The Lamu Port-South Sudan-Ethiopia-Transport (LAPSSET) corridor transport and infrastructure project runs along the eastern edge of the Tana River Basin, with parts of the railway line and main road passing through the basin. The railway line continues through the high-potential agricultural land in the north of the basin on to Nairobi.

14% of projected refugia (around 10,500 km^2^) with 2°C of warming which has not already been converted to agriculture are at risk from the activities described in the National Spatial Plan (Table 1). Refugia for at least one taxon that are at risk from proposed development are also shown on Fig 4. This includes around 1700 km^2^ of projected refugia inside protected areas, including the Meru National Park. The greatest threat comes from proposed irrigation areas (9% of the refugia at risk). 31% of projected refugia with 4.5°C warming, which have not already been converted to agriculture, are threatened by the National Spatial Plan. Again, the greatest area is threatened by proposed irrigation (21% of projected refugia). With 4.5°C warming, all of the refugia not threatened by irrigation development or already converted to agriculture are only classified as refugia for amphibians (S1 Fig). This suggests that plants, mammals, birds and reptiles could be more at risk from these development plans.

**Table 1:** Projected refugia at risk from National Spatial Plan activities.

	With 2°C warming			With 4.5°C warming		
NSP Activity	Area (sq km)	% of refugia	% of basin	Area (sq km)	% of refugia	% of basin
Proposed irrigation	6,981	9	7	4,274	21	5
Proposed economic growth areas	3,148	4	3	2,791	14	3
High agricultural potential land	1,193	2	1	126	1	0
Medium agricultural potential land	3,434	4	4	1,634	8	2
All activities ^a^	10,586	14	11	6,281	31	7

The area of projected refugia for at least one taxonomic group within the Tana River Basin with 2°C warming and 4.5°C warming that is at risk from activities proposed within the National Spatial Plan (NSP) [53]. This table refers to areas that have not already been converted to agriculture. Some NSP activities overlap with one another.

^a^ This includes all proposed agricultural, irrigation and economic expansion areas. Some of which overlap with one another spatially.

### Potential areas for restoration

**Fig 5:**
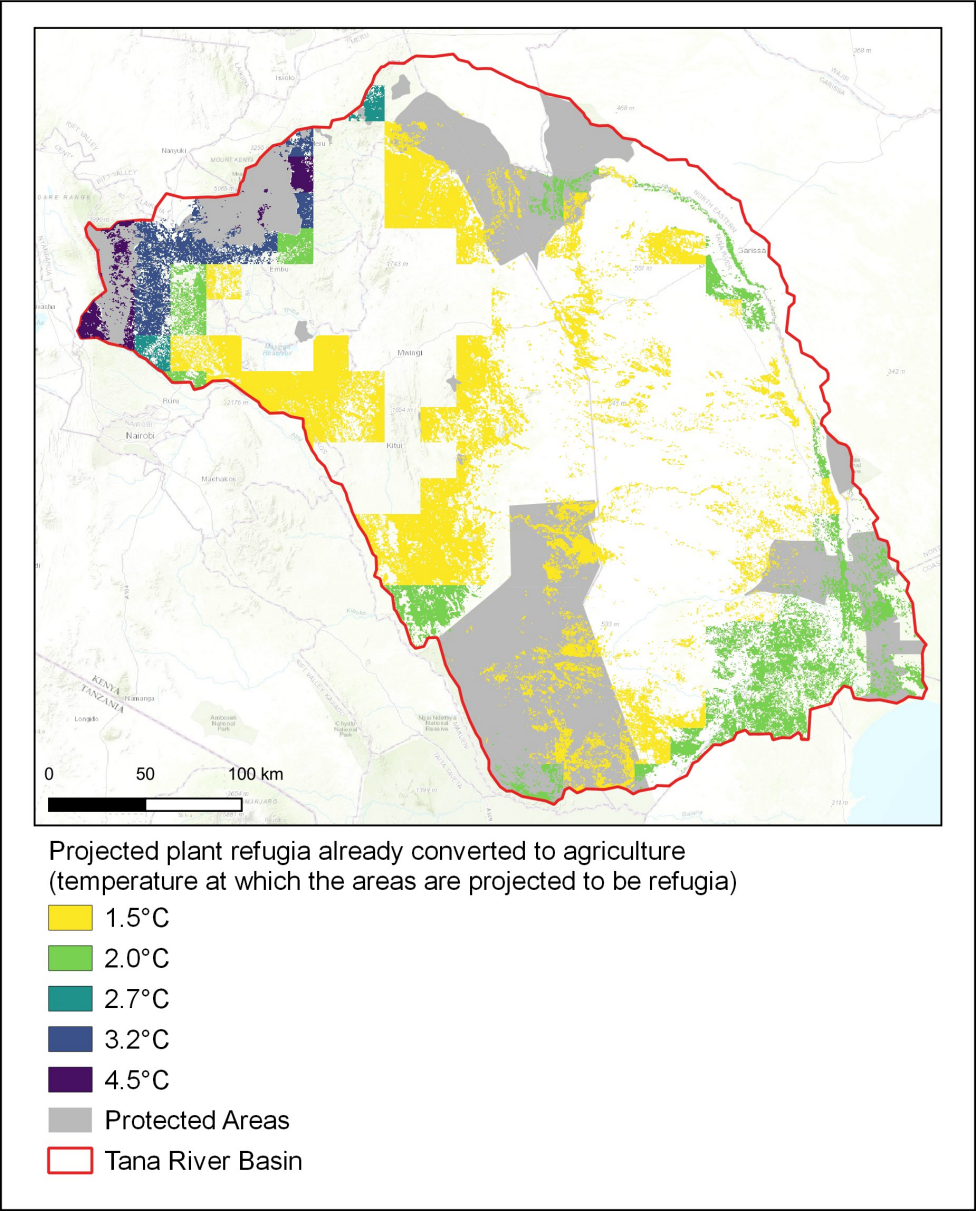
Agricultural land that was projected to be refugia for plants under different levels of warming. These are areas that are projected to contain refugia for plants which have already been converted to agricultural land uses. Projected refugia are identified using the criterion that 11 or more GCMs agree on their presence.

The land projected to contain refugia for plants but converted to agriculture would be areas to prioritise for restoration, contributing to Kenya’s post-2020 biodiversity and restoration targets. Fig 4 showed the projected refugia with 2°C and 4.5°C for at least one taxonomic group that have been converted to agriculture, compared to other categories of refugia within the basin. By contrast, Fig 5 shows projected refugia for plants with all levels of warming that have been lost to agriculture. Agricultural lands that were projected to be refugia for plants with 1.5°C global mean temperature rise are spread throughout the basin, covering 23% of the total area. If warming is limited to this level, a greater proportion of agricultural land within the basin could be restored to benefit biodiversity, act as carbon sinks, and provide additional ecosystem services. Significantly less agricultural land was projected to have the potential to act as refugia for plants with 2°C warming (8.7% of the basin) or greater, so the refugial area available for restoration is reduced. Refugia for plants are concentrated in the high elevations in the north and delta region in the south, as well as along the main Tana River in the east. With higher levels of warming, the agricultural land which could be restored is confined to the upland areas in the north of the basin. Much of this land borders PAs, including the Mount Kenya and Aberdare National Parks. Very little agricultural land in the basin was projected to be refugia with 4.5°C warming, covering only 0.7% of the total area of the basin. This land would be the most beneficial to restore as it is likely to protect biodiversity even if warming is not limited to levels agreed in the Paris Agreement or through INDCs.

### Importance of facilitating movement

Ensuring species are able to move freely across the Tana River Basin–to shift with their preferred climate envelope–is likely to help preserve biodiversity in a changing climate. The differences in species richness for mammals and birds when dispersal is included compared to when species are unable to shift their ranges outside of their current climate envelope are greatest in the northeast of the basin (S9–S12 Figs). The differences between the two scenarios are more pronounced when temperatures reach 4.5°C above pre-industrial levels (S2 and S4 Figs), showing the importance of allowing species to adapt in scenarios with high global temperatures.

**Fig 6:**
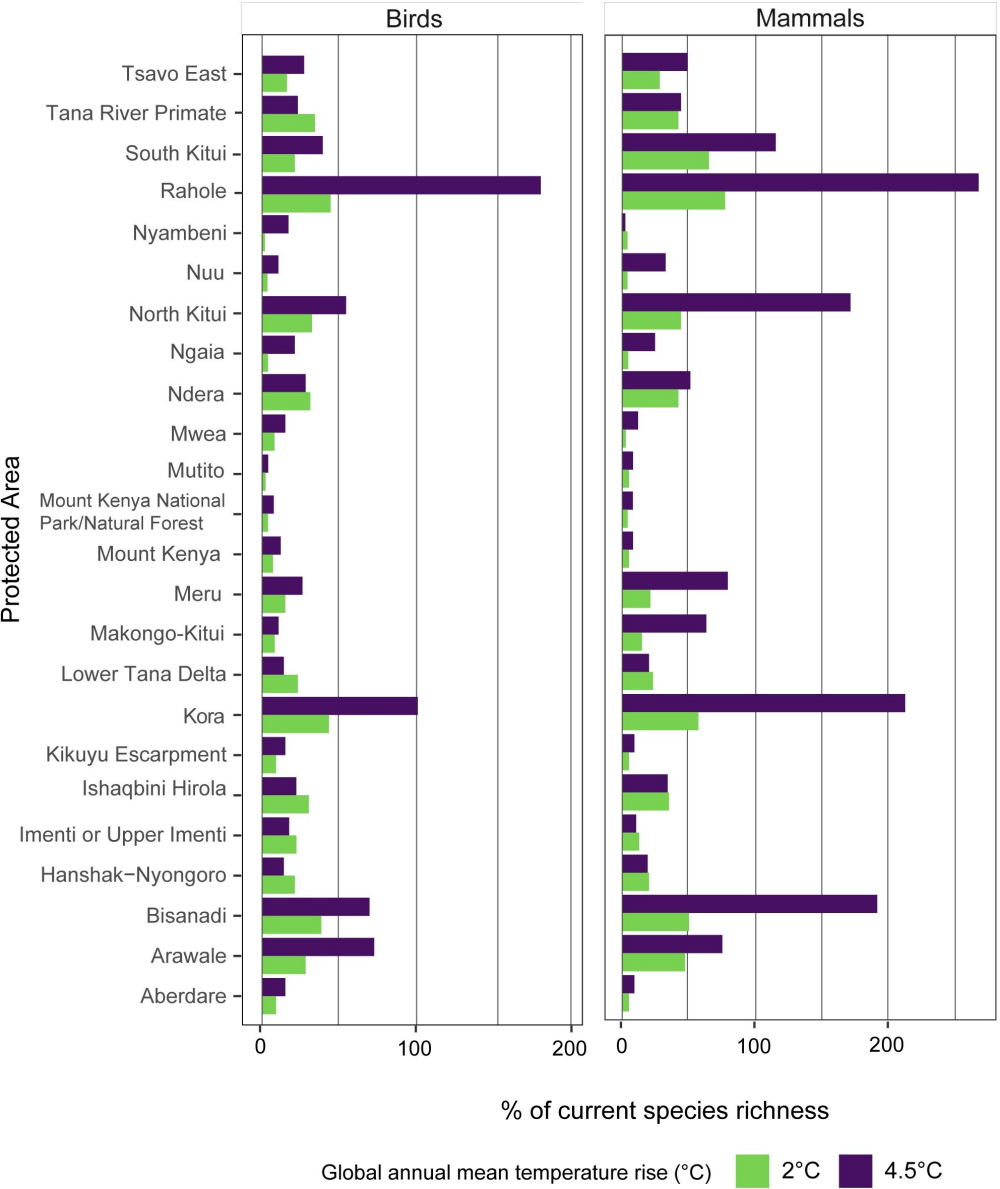
Additional percentage of current species richness with dispersal. This figure shows the difference between the proportion of species richness remaining in each PA without dispersal and when realistic dispersal rates are included, with 2°C and 4.5°C global temperature rise. Large values indicate new species have moved into the PA.

The importance of facilitating movement in terms of avoided loss of species richness is shown in Fig 6 for birds and mammals. Although these PAs may not be refugia for the existing taxa, they are still important for protecting biodiversity, especially that which is moving in response to a changing climate Allowing species to adapt to the changing conditions by dispersal could protect a greater proportion of birds and mammals in all PAs. When species are able to move freely to track their preferred climatic conditions, Rahole National Reserve and Kora National Park become extremely important for both birds and mammals with the higher levels of warming. The same is shown for North Kitui and Bisanadi National Reserves for mammals. An increase in bird and mammal species richness compared to current modelled levels is projected for these PAs.

## Discussion

### Implications of climate change for species richness

The five taxa examined here have been shown to be at risk from climate change, with higher temperatures leading to greater impacts. Large proportions of the current species richness could be lost from the basin with higher levels of warming. There are clear benefits of mitigation (i.e. the reduction of GHG emissions) to preserving the biodiversity of the Tana River Basin. The dangers of higher warming to biodiversity in terms of climatic range loss have previously been discussed [12, 47, 51]. Limiting global temperature rise to 2°C could avoid around 60% of global climatic range loss compared to higher (3.6–4°C) warming [13]. A trait-based assessment of birds, amphibians and corals found that large proportions were highly vulnerable to 2°C of warming [12]. All PAs analysed here have been shown to benefit from mitigation. However, as sizeable losses are still projected when warming is constrained to 2°C above pre-industrial levels, it is clear that adaptation is also important, as well as the achievement of the true goal of the Paris Agreement to limit global warming to ‘well below 2°C’ and to ‘pursue efforts’ to limit warming to 1.5°C.

The benefits of limiting warming to as close to this 1.5°C level as possible for the biodiversity of the basin are clear. There is a substantial difference between the proportion of the basin that is projected to contain refugia for plants between 1.5 and 2°C warming.

The benefits of allowing species to track their preferred climate space are clearly shown in the results for mammals and birds. This was also noted in a global analysis [51], which considered the effects of different levels of warming and dispersal scenarios on WWF priority places [41].

The rate of climate warming will also have implications for biodiversity. If temperature thresholds are crossed early, few species will have the time to disperse and adapt. Birds and mammals tend to see the greatest population declines in areas that experienced the most rapid warming, with the relationship stronger for birds [56]. However, it is important to remember that more data typically exists for these taxa than others, such as reptiles and amphibians. If natural dispersal does not occur at a sufficient rate, wildlife corridors may not be effective in preserving the species [57]. In addition, decision-makers will have less time to facilitate movement, for example by expanding PAs or developing corridors [47]. A slower rate of warming allows greater time for such conservation action, as well as for the natural processes of dispersal and even (for some species) evolutionary change [58]. This further demonstrates the importance of mitigation for preserving biodiversity, since mitigation ‘buys time’ for adaptation.

### Projected climate refugia

Refugia are projected within the Tana River Basin, demonstrating the importance of protecting the area. Even with high levels of warming (4.5°C), projected refugia are still identified within the mountains in the north of the basin, as well as in the Tana Delta region in the southeast. For amphibians, there is a shift in the location of refugia in the north of the basin with different levels of warming. It is likely that this is due to changes to rainfall projections between the scenarios. Within the Wallace Initiative, RCP 2.6 in the 2080s is a proxy for a 2°C world and RCP 8.5 in the 2080s is a proxy for the 4.5°C world. Under RCP8.5 conditions, precipitation across the Tana River Basin is projected to increase substantially compared to RCP2.6, particularly in the north of the basin where these cells are located [59, 60]. Amphibians are affected by limits on precipitation and water availability [61]. These cells are projected to be refugia with 4.5°C warming due to the increase in precipitation projected in this area of the basin. When no taxonomic group is able to shift their range in response to warming, more cells are projected to act as refugia for animals than for plants. This shows that plants could be particularly vulnerable to the changes in climate. Changes to the plants of the Tana River Basin are likely to have compound effects on the animals that depend on them for food and shelter. The difference between the costs of conserving biodiversity and global spending on conservation [62] necessitates prioritisation of spending. Identifying likely climate refugia within the basin may assist with this prioritisation.

### Implications for PAs

A key consideration is whether the PAs will be sufficient for protecting biodiversity in a changing climate. These results show that, with higher temperatures, the current PAs within the Tana River Basin could be insufficient for protecting many species. PAs form the first defence for biodiversity against many human activities and are the cornerstone of *in situ* biodiversity conservation. There is evidence of biodiversity declines within protected areas around the world [63, 64], but species are generally better protected inside PAs than outside in the wider landscape [65]. This study has shown that many of the PAs in the basin already have a significant proportion of land within them turned over to agriculture. At the national level, it has been estimated that, as of 2015, 7.2% of land within PAs in Kenya had been converted to anthropogenic uses [66].

Many PAs in East Africa were originally established as game reserves and converted to government-owned national parks following independence [66, 67]. These PAs were designated to protect large mammal populations or particular charismatic species rather than truly conserving the full biodiversity of the area. These PAs do not adequately cover the hotspots of vertebrate endemism [66], so it is somewhat unsurprising that they may be insufficient for protecting biodiversity in a changing climate.

Maintaining connectivity within landscapes, particularly between conservation areas, is vital for reducing pressure on ecosystems and encouraging demographic links and gene flow [68]. This may involve the creation of new protected areas that better protect a greater range of species. However, several studies have noted that land available for the creation of new PAs in East Africa is extremely limited [66, 69], so restoring previously converted land is also likely to be necessary to better protect biodiversity.

Alternatively, the land projected to contain refugia that has not previously been converted to agricultural uses could be managed sustainably (i.e. not converted to urban or cropland through the NSP, or overgrazed) rather than gazetted as strict nature reserves as creating new strict PAs sometimes leads to additional problems, such as those arising from mismanagement, negative local attitudes towards conservation and fences creating additional barriers to movement, which will undermine their effectiveness for conservation [70]. In Kenya, threats to biodiversity within PAs come from a range of activities, including increased poaching, human encroachment, over-exploitation of natural resources by communities living within the PA boundaries, negative impacts associated with the expansion of tourism after the area is protected, and perimeter fencing interfering with wildlife movement [71]. However, the management of protected areas must consider the needs of different stakeholders as well as the ecological value of the sites. There are many ways in which an area can be protected and many of these have the potential to provide equal benefit to biodiversity. PAs that involve local communities as stakeholders have been found to produce better conservation outcomes than nationally designated protected areas [72, 73]. Land must be managed sustainably, using appropriate rangeland strategies like the observance of carrying capacity and conservation of vegetation [53], to avoid land degradation from overgrazing.

### Implications of development plans for biodiversity

Agricultural expansion (including irrigation) is a major feature of the National Spatial Plan. This also presents a significant threat to climate refugia as many areas earmarked for agricultural expansion have been shown to contain refugia for biodiversity. A significant proportion (29% of refugia with 2°C warming, 23% of refugia with 4.5°C warming) have already been converted to agriculture. As this part of the analysis was based on agricultural data from 2015, it is likely that additional areas have been converted since then; further limiting the proportion of projected refugia available for species in the future.

Further losses to the area of refugia as a result of activities in the NSP would be damaging to the future of biodiversity within the basin. Projected refugia for both animals and plants overlap with proposed irrigation areas, high- and medium-potential agricultural land and economic growth areas from the NSP. This may lead to trade-offs between agricultural or urban development and biodiversity protection in some regions of the basin. Reducing the activities in the NSP (agricultural, irrigation and economic expansion areas) by 24% could remove the threat to refugia with 2°C warming while reducing the activities by only 14% could remove the threat to areas that are still projected to contain refugia with 4.5°C warming. There are also benefits to agriculture of retaining refugia alongside NSP activities, such as the importance of these natural areas for supporting pollinators and the subsequent increase in pollination services for crops. Detailed spatial planning at the local level should take these co-benefits into account, incorporating conservation areas into wider agricultural landscapes.

Although it was not possible to quantify the effects, other elements of the National Spatial Plan will threaten biodiversity. The LAPSSET corridor railway line and dam construction may restrict the movement of species towards the higher elevations. The importance of facilitating movement has been clearly demonstrated in the results for birds and mammals. Building additional dams along the upper reaches of the Tana River and its tributaries may prevent species from moving to more suitable climates. In addition, dam construction upstream will have effects on the whole ecosystem, including the PAs. Changes to land use and hydrological properties of unprotected parts of ecosystems can alter the biodiversity and ecosystem functioning within PAs [74]. So, even if existing PAs are preserved within the larger agricultural or economic development areas, the effects of intensive land uses on the edge of the PAs may limit their suitability for wildlife.

However, the interactions between land use and climate and their combined effects on biodiversity are very complex and cannot all be considered within this research. Understanding all of these possible interactions is an ongoing area of research [75].

From this analysis, it is clear that there will be hotspots of conflict between competing land uses within the Tana River Basin. The Upper Tana Basin is likely to be an area of trade-offs. As the climate warms, the land further upslope is likely to become more and more suitable for plants and animals. A large range of species will be forced to occupy this smaller space. The Mount Kenya National Park and Natural Forest and Aberdare National Park are important PA in the north of the basin that are projected refugia for plants and animals under high levels of warming. These refugia are most at risk as they are considered high potential agricultural land. Preventing expansion of agriculture in these areas (around 125 km^2^, less than 0.01% of the area of activities proposed in the National Spatial Plan) could protect areas projected to contain refugia with the highest warming levels in the north of the basin.

Another geographical region that may experience trade-offs is the Tana Delta. In the Delta, the Lower Tana Delta Conservation Trust, Witu Forest Reserve and the Hanshak-Nyongoro Community Conservancy are important PAs that are also projected to be refugia for a range of species under high levels of warming. The Basra Reed Warbler (*Acrocephalus griseldis*), for example, overwinters in the Tana River Delta and so is also threatened by the large-scale agriculture projects planned for the area [76]. Without PAs in this delta region being suitable for the species, the reed warbler may not be able to overwinter in the basin. It is helped a little if dispersal is feasible but limiting warming (mitigation) would be particularly beneficial for this species. This area is already highlighted as a hotspot of human-wildlife conflict [77]. Development plans and refugia for biodiversity in the delta region are likely to exacerbate this conflict in the future. The policymakers aim to establish more community conservancies around the Tana Delta development to protect wildlife, but many wildlife routes in this area are currently considered as blocked [77], suggesting that species will find it difficult to disperse and adapt to the changing climate and land uses.

The changing climate may undermine the development activities, making some land use changes unsustainable and inappropriate. The GoK has identified climate change as a significant challenge to attaining the Vision 2030. However, to date, there is little direct consideration of climate change in existing sectoral development plans.

### Adaptation options

Based on these results, various recommendations for adaptation can be determined. First, improving the connectivity of the PAs would be extremely important to facilitate species’ movement. Maintaining or improving corridors is generally considered to be a better adaptation choice than other options, such as assisted colonisation (also known as managed relocation). Wildlife corridors are seen as lower risk and reduce the possibility of invasive species problems [78]. Facilitating dispersal may involve restoring or protecting the habitats between existing PAs; therefore encouraging species to move into areas that become climatically suitable. However, if corridors are not sufficient or if the rate of warming is too fast for species to keep up, assisted colonisation may become necessary to preserve some species.

Harmful development and land uses on the edge of reserves could disturb the wildlife. This could prove a particular problem for the small PAs, such as the forest reserves. Extremely small PAs are unlikely to maintain sufficient genetic diversity to fully protect the species within them. Small PAs within larger unprotected ecosystems are some of the most vulnerable [79]. As land use changes occur, and the reserves become increasingly isolated through a loss of landscape connectivity, the species present may suffer. Enlarging some of the smallest PAs may help conserve biodiversity. In addition, creating buffer zones of partially restricted land around the PAs may prevent the encroachment of harmful land uses which may affect the conservation inside the parks [80]. However, the effectiveness of this method has been questioned. The concept is used in South Africa to protect land around the edge of PAs where the biodiversity, hydrology or aesthetics of the park could be affected by development activities [81].

Furthermore, the biodiversity of the Tana River Basin would benefit from the better regulation of the PAs both now and in the future. There are still problems of deforestation and livestock within the PAs [36, 82], and, as shown in the results, cropland is present in all PAs to some extent. In practice, biodiversity conservation and adaptation are likely to involve a range of these measures, which are known as integrated conservation strategies. Biodiversity adaptation measures are generally compatible with other adaptation strategies, except where the requirements of one species are in opposition to those of another species of conservation concern. Therefore, these measures can be considered low risk. These are options which provide benefits regardless of the uncertainties in the climate change projections [83]. The sizeable impacts on biodiversity of the Tana River Basin, even in a situation when the Paris Agreement global temperature targets are met, shown here demonstrate the urgency of the problem and show that is paramount that the GoK start considering climate change in the policies now rather than planning to do so in the future. Existing policies and plans should be re-evaluated with the effects of climate change borne in mind.

### Implications for restoration

Protecting biodiversity has the potential to contribute to other environmental goals, including ecosystem restoration targets. In order to account for climate change projections, restoration efforts should focus on areas of projected refugia which have already been converted to other land uses. These areas are projected to remain climatically suitable for most of the species present in the area prior to conversion to agriculture, some of which may persist within the agricultural landscape. Therefore, the benefits of these refugia may not be entirely lost, which may facilitate ecological restoration or the implementation of sustainable practises to better protect biodiversity. Particular attention should be paid to areas that border current protected areas, as restoring these lands may increase habitat connectivity and facilitate dispersal, and projected refugia, where species will be more likely to persist in a changing climate. Many of these areas are in the highlands in the northwest of the basin. Restoring these slopes ties in with the GoK’s targets of protecting the Water Towers [84, 85]. Kenya’s Bonn Challenge commitments include a total restoration target of 4,210,000 hectares, including all degraded forests and riverine vegetation. This strategy includes growing over 7 billion trees by 2030 [84]. There is also significant restoration potential within the PAs of the Tana River Basin. Restoring degraded land to ensure biodiversity protection could also lead to synergistic benefits, such as increasing the supply of ecosystem services, such as pollination services and carbon storage and sequestration.

Restoration will lead to trade-offs between agricultural development and biodiversity protection or forest regeneration. Around 80% of Kenya is arid or semi-arid land (ASAL), so there is a limited amount of land available for natural habitats and agriculture. The restoration of land which is currently used for agriculture can lead to increased pressure in other areas. Therefore, restoration should be carefully planned at the basin or country scale in order to minimise negative effects across the whole area. Abandoned agricultural land should be a priority for restoration. Within the Tana River Basin, several large-scale agricultural schemes have become abandoned due to low yields and climate variability. This land could be restored without significantly altering the economy of the basin.

However, as the area projected to contain refugia decreases with greater levels of warming, so does the restoration potential. Areas not considered to be refugia, by definition, are expected to become climatically unsuitable for at least 25% of current species. Therefore, many of the species present in the area prior to conversion to agriculture may be unable to tolerate the new climatic conditions if higher levels of warming are realised. The projected reduction in plant species richness may undermine efforts towards achieving these restoration targets. The Kenya Forestry Research Institute [86] compiled a list of suitable tree species for afforestation or reforestation projects. However, many of these species have been projected to see a reduction in climate suitability with higher temperatures [60]. Some species identified as less sensitive to climate changes are the neem tree (*Azadirachta indica*), sycamore fig (*Ficus sycomorus*) and wild date palm (*Phoenix reclinata*). Future research into additional tree species with less sensitivity to the changing climate which can be used in ecosystem restoration projects is vital. In terms of restoration, there are still gaps in knowledge of how to effectively restore most types of ecosystems [87]. Some researchers have shown the importance of intact faunal communities, particularly those that disperse seeds, for the successful restoration of forest areas [88].

### Implications for tourism

These changes to the biodiversity of the Tana River Basin will have implications for tourism. Kenya is known internationally as a hotspot of wildlife tourism, with the majority of tourists visiting to watch wildlife. Nearly 10% of Kenya’s GDP comes from tourism. Most visitors focus on the more well-known PAs in the south of the country. Visitor numbers in selected PAs in South Africa, Namibia and Botswana were higher in areas where there was high predator species richness and a presence of locally rare ungulate species [89]. A high diversity of large mammals was also found to be important in attracting high numbers of wildlife tourists. The PAs within the Tana River Basin generally saw a decrease in suitability for many of the predator species examined here, such as the wild dog, cheetah and African lion. Similarly, a reduction in the number of species in the PAs with climate change could impact tourist numbers. This could be particularly significant for the Tsavo East National Park, which could experience reductions in the number of species present in the future. Other than the Masai Mara, the Tsavo ecosystem is the most popular with tourists [77]. In order to maintain high visitor numbers, additional conservation spending may be necessary.

### Limitations

Although this analysis provides important information about the impacts of climate change and development plans on the terrestrial biodiversity of the Tana River Basin, a number of caveats need to be considered. There are several relevant factors that have not been included in this analysis due to the spatial scale used in the Wallace Initiative database [47]. These factors include the potential spread of disease pathogens and pests, interactions between species (food availability, predator-prey relationships and competitive interactions) [90], the effects of extreme climatic events [91] and the direct biotic effects of increases in CO_2_ concentrations on plants [92]. In addition, many species, especially narrow-ranged endemic species, are not included in the Wallace Initiative database. Narrow-ranged species are generally sensitive to climate change [93] so many of the endemic species not included in this analysis will also be vulnerable to the effects of global temperature rise. Species with fewer than 10 data points (occupied grid cells) were excluded from the Wallace Initiative database in order to maintain a robust analysis [47].

Despite these limitations, the Wallace Initiative fulfils many of the criteria recently set out as contributing to best practice in species distribution modelling [94]. GBIF data combines many datasets worldwide and the data was further cleaned (checked for locational consistency and outliers) before use. Commission errors were accounted for by clipping species to their biogeographic zones and omission errors were taken into account by generous buffers around species distributions. Furthermore, uncertainty is taken into account by considering a range of GCMs and dispersal rates.

There are some general limitations with species distribution models which should also be considered. In some cases, there is not sufficient data available to fully inform the model as to the true distribution of a species [95, 96]. Datasets used to drive the SDM are often biased because of an unequal sampling effort across the study area [97]. SDMs can be overfitted, which can lead to flawed outputs by limiting the model’s capacity to generalise. During the development of the Wallace Initiative, a reduced set of variables was used to minimize potential autocorrelation. SDMs cannot include and account for all biotic and abiotic factors. Even though various uncertainties exist, SDMs are extremely useful for examining the future impacts of climate change on species. This knowledge is fundamental for policymakers and conservation planners.

For this analysis, it was assumed that all of the species that had suitable climate space within the PAs with higher temperatures are protected in these spaces and occur in viable populations. Although this might not be the case, this assumption allows for the identification of species that require more future conservation attention (i.e. those not occurring at all within the PAs). Furthermore, some PAs extend beyond the basin. For instance, the Tsavo East National Park and it’s connected Tsavo West and Amboseli National Parks. The greater Tsavo ecosystem may contain refugia even though the part of the Tsavo East National Park located within the Tana River Basin may not. Even if this is the case, it further demonstrates the importance of allowing species to disperse across the landscape.

## Conclusions

This study showed significant implications of projected climate change on the terrestrial biodiversity of the Tana River Basin. When these changes in species distributions are considered alongside development plans, it becomes clear that trade-offs between development and biodiversity protection may occur in the future. Various adaptation options for biodiversity have been proposed, including expanding protected areas to better conserve projected climate refugia. Restoring lands that are projected to be refugia for plants but have already been converted to agriculture could help conserve biodiversity, facilitate species movements and contribute to Kenya’s ecosystem restoration targets. By understanding these potential conflicts between biodiversity and development, decision makers can ensure the basin is managed in a way as to ensure effective future biodiversity protection, successful ecosystem restoration and sustainable socio-economic development within this important area of Kenya.

## Supporting information

S1 Table Protected Areas (PAs) within the Tana River Basin. Data on the locations of protected areas were obtained from the World Database on Protected Areas (WDPA). This table only includes the PAs considered in this study.
(DOCX)

S1 Fig Locations of projected refugia for amphibians with 2°C and 4.5°C warming. A refugium is identified in a grid cell only if at least 11 of the 21 GCMs agree in projecting its existence.
(TIF)

S2 Fig Locations of projected refugia for birds with 2°C and 4.5°C warming. A refugium is identified in a grid cell only if at least 11 of the 21 GCMs agree in projecting its existence.
(TIF)

S3 Fig Locations of projected refugia for mammals with 2°C and 4.5°C warming. A refugium is identified in a grid cell only if at least 11 of the 21 GCMs agree in projecting its existence.
(TIF)

S4 Fig Locations of projected refugia for plants with 2°C and 4.5°C warming. A refugium is identified in a grid cell only if at least 11 of the 21 GCMs agree in projecting its existence.
(TIF)

S5 Fig Locations of projected refugia for reptiles with 2°C and 4.5°C warming. A refugium is identified in a grid cell only if at least 11 of the 21 GCMs agree in projecting its existence.
(TIF)

S6 Fig Locations of projected refugia for all five taxa with 2°C and 4.5°C warming. A refugium is identified in a grid cell only if at least 11 of the 21 GCMs agree in projecting its existence.
(TIF)

S7 Fig GCM agreement that protected areas contain refugia with different levels of warming. These results are based on a no dispersal scenario. The five classes of species are amphibians, birds, mammals, plants and reptiles.
(TIF)

S8 Fig Key features of Kenya’s National Spatial Plan affecting the Tana River Basin. Key features of the National Spatial Plan (Government of Kenya, 2017) within the Tana River Basin boundary were digitised using GIS.
(TIF)

S9 Fig Importance of facilitating dispersal for birds with 2°C warming. The first column (left) shows species richness remaining when species are not able to shift their ranges, the centre column shows the scenario where species are able to disperse, and the final column (right) shows the difference between the first two. The top panels show the 10^th^ percentile, the middle shows the 50^th^ percentile and the bottom panel shows the 90^th^ percentile.
(TIF)

S10 Fig Importance of facilitating dispersal for birds with 4.5°C warming. The first column (left) shows species richness remaining when species are not able to shift their ranges, the centre column shows the scenario where species are able to disperse, and the final column (right) shows the difference between the first two. The top panels show the 10^th^ percentile, the middle shows the 50^th^ percentile and the bottom panel shows the 90^th^ percentile.
(TIF)

S11 Fig Importance of facilitating dispersal for mammals with 2°C warming. The first column (left) shows species richness remaining when species are not able to shift their ranges, the centre column shows the scenario where species are able to disperse, and the final column (right) shows the difference between the first two. The top panels show the 10^th^ percentile, the middle shows the 50^th^ percentile and the bottom panel shows the 90^th^ percentile.
(TIF)

S12 Fig Importance of facilitating dispersal for mammals with 4.5°C warming. The first column (left) shows species richness remaining when species are not able to shift their ranges, the centre column shows the scenario where species are able to disperse, and the final column (right) shows the difference between the first two. The top panels show the 10^th^ percentile, the middle shows the 50^th^ percentile and the bottom panel shows the 90^th^ percentile.
(TIF)
