# Supplementary material for: Addressing risks to biodiversity arising from a changing climate: The need for ecosystem restoration in the Tana River Basin, Kenya
Source: PLoS One. 2021 Jul 21;16(7):e0254879. doi: 10.1371/journal.pone.0254879 (PMC8294490; doi:10.1371/journal.pone.0254879)
Supplement: S1 Table — Data on the locations of protected areas were obtained from the World Database on Protected Areas (WDPA). This table only includes the PAs considered in this study. (DOCX) [file pone.0254879.s001.docx]

**Supporting information**

**S1 Table****. Protected Areas (PAs) within the Tana River Basin.** Data on the locations of protected areas were obtained from the World Database on Protected Areas (WDPA). This table only includes the PAs used in this study.

| **NAME** | **DESIGNATION** | **Area (km^2^)** |
| --- | --- | --- |
| Tsavo East | National Park | 11747 |
| Mount Kenya National Park/Natural Forest | World Heritage Site | 2023 |
| Mount Kenya | Forest Reserve | 2010 |
| South Kitui | National Reserve | 1833 |
| Kora | National Park | 1788 |
| Rahole | National Reserve | 1270 |
| Ndera Community Conservancy | Community Nature Reserve | 1155 |
| Meru | National Park | 870 |
| Hanshak-Nyongoro Community Conservancy | Community Nature Reserve | 792 |
| Aberdare | National Park | 766 |
| North Kitui | National Reserve | 745 |
| Ishaqbini Hirola Community Conservancy | Community Nature Reserve | 732 |
| Bisanadi | National Reserve | 606 |
| Arawale | National Reserve | 533 |
| Lower Tana Delta Conservation Trust | Community Nature Reserve | 512 |
| Kikuyu Escarpment | Forest Reserve | 376 |
| Solio Ranch and Rhino Sanctuary | Private Ranch | 200 |
| Tana River Primate | National Reserve | 169 |
| Imenti or Upper Imenti | Forest Reserve | 122 |
| Ngaia | Forest Reserve | 82 |
| Mwea | National Reserve | 68 |
| Nyambeni | Forest Reserve | 52 |
| Nuu | Forest Reserve | 45 |
| Makongo-Kitui | Forest Reserve | 32 |
| Mutito | Forest Reserve | 30 |
