# Supplementary figures and images for: Addressing risks to biodiversity arising from a changing climate: The need for ecosystem restoration in the Tana River Basin, Kenya
Source: PLoS One. 2021 Jul 21;16(7):e0254879. doi: 10.1371/journal.pone.0254879 (PMC8294490; doi:10.1371/journal.pone.0254879)

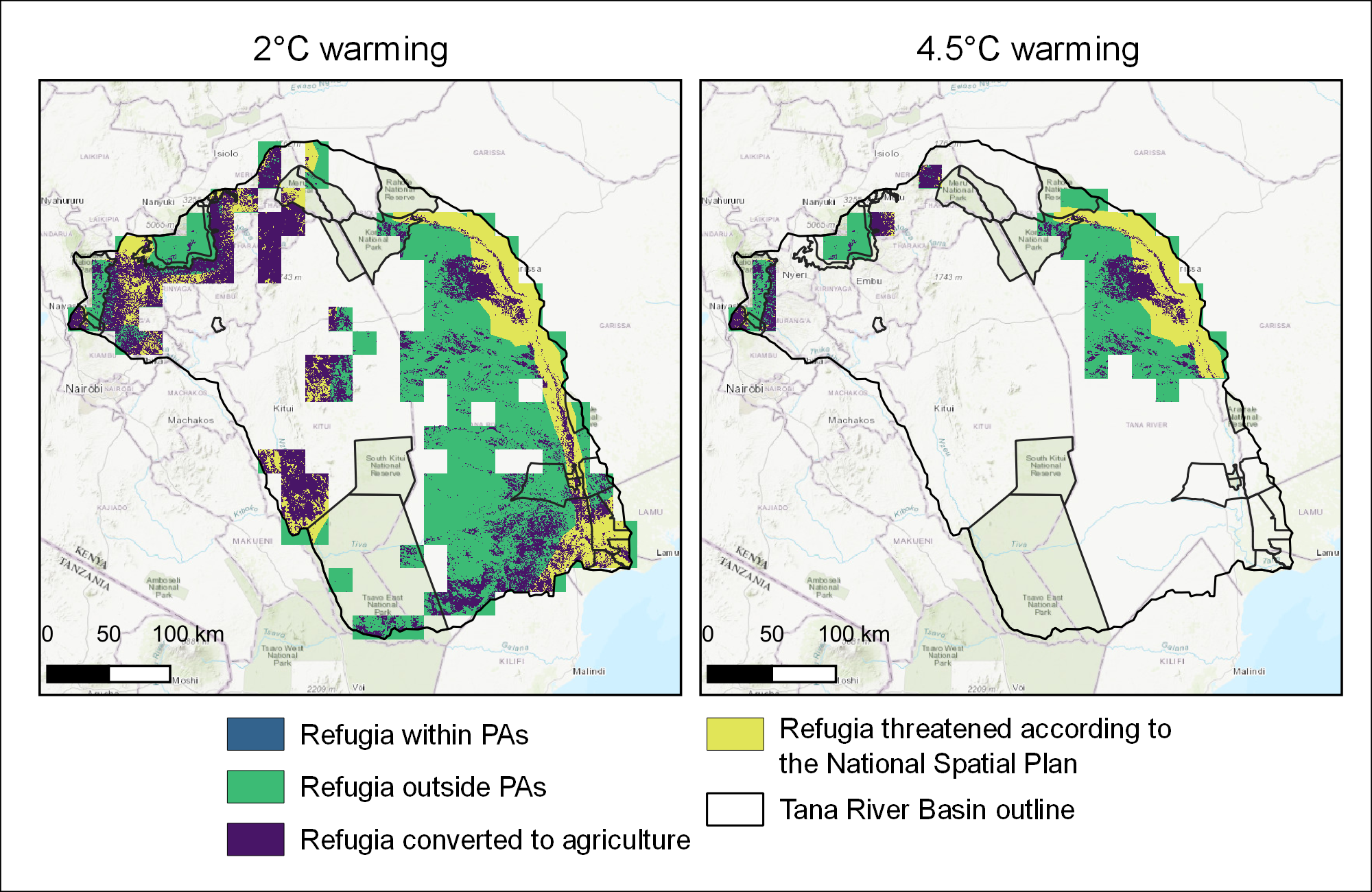

Supplement: S1 Fig — A refugium is identified in a grid cell only if at least 11 of the 21 GCMs agree in projecting its existence. (TIF) [file pone.0254879.s002.tif]

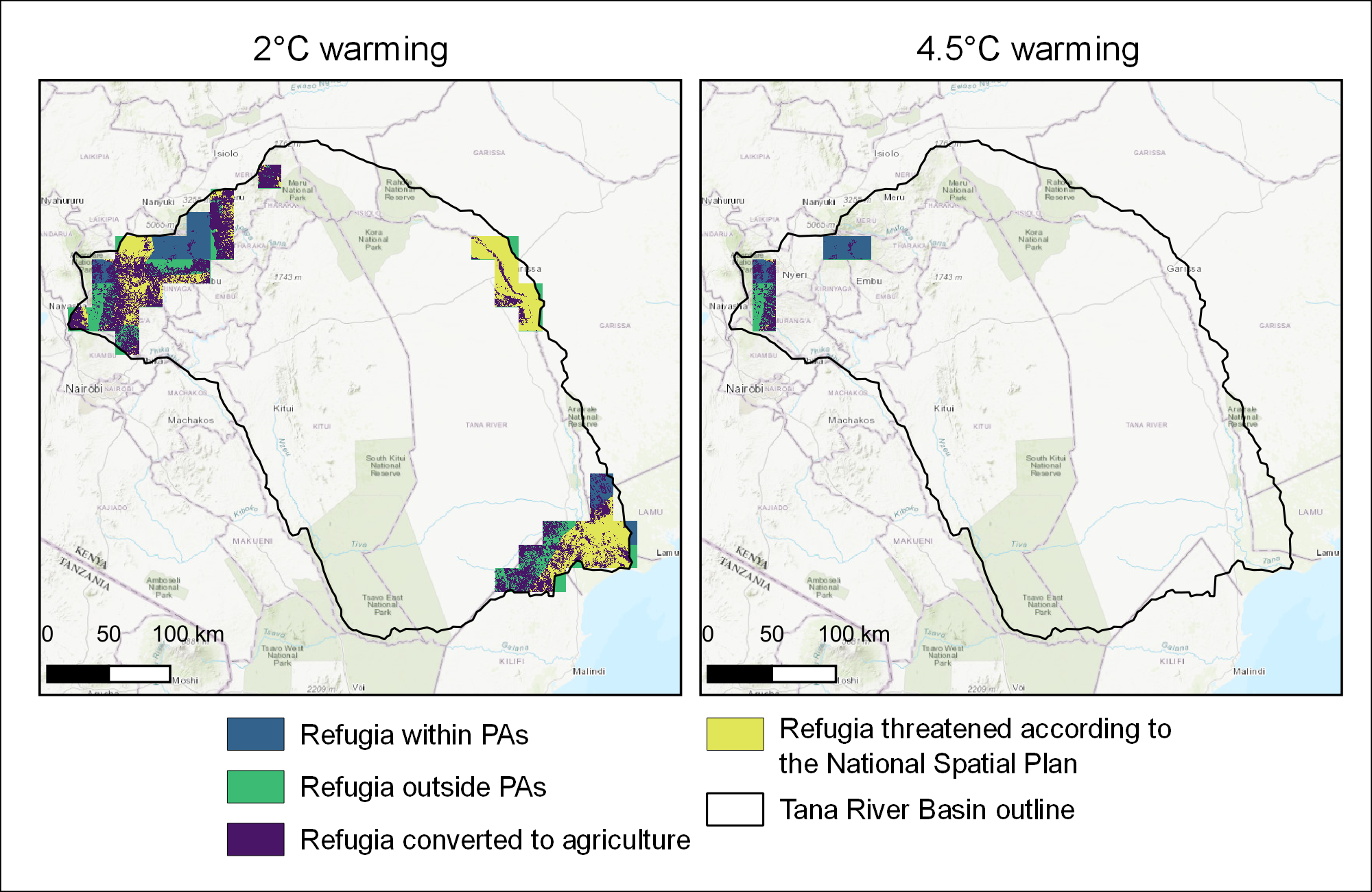

Supplement: S2 Fig — A refugium is identified in a grid cell only if at least 11 of the 21 GCMs agree in projecting its existence. (TIF) [file pone.0254879.s003.tif]

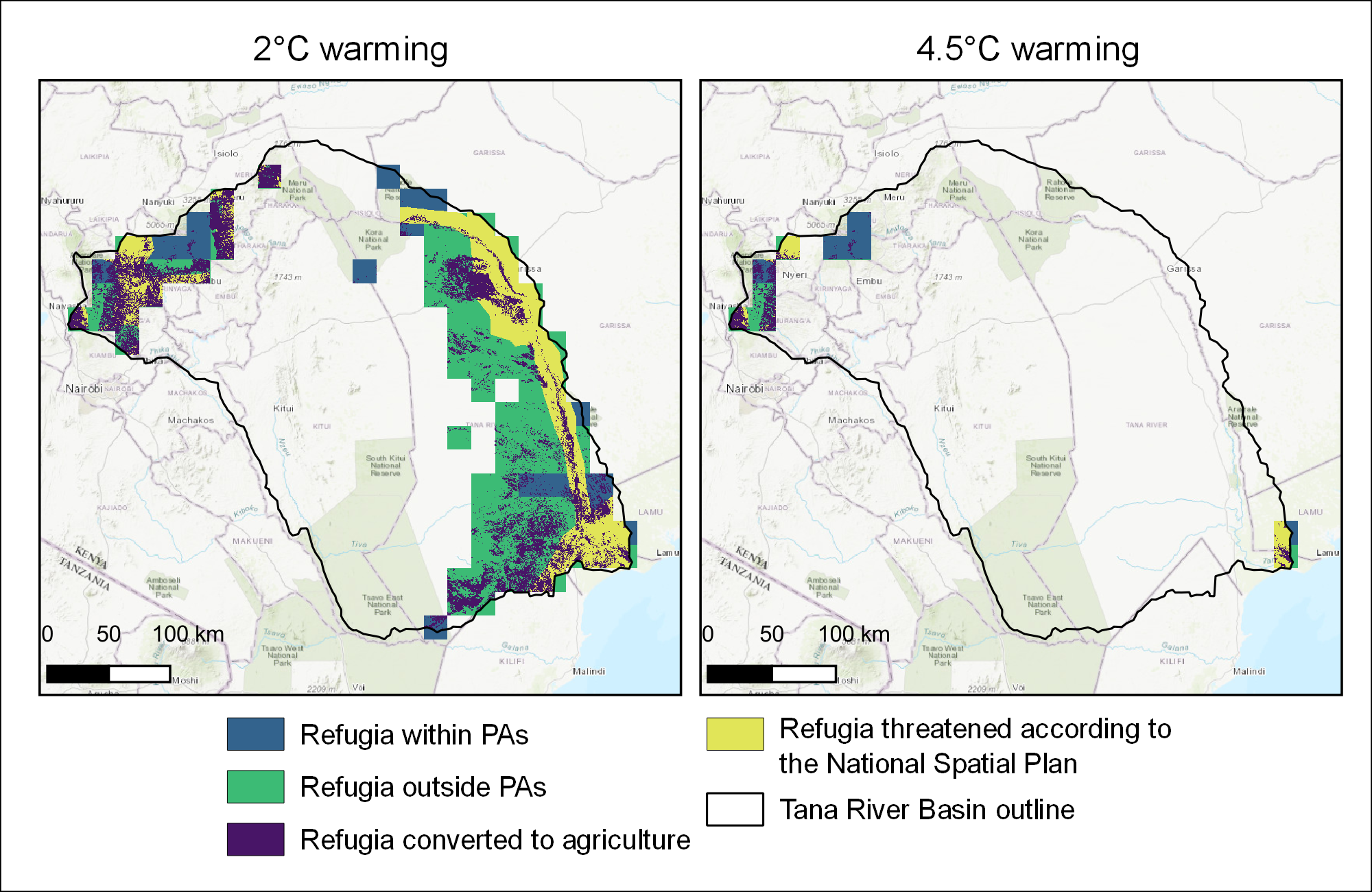

Supplement: S3 Fig — A refugium is identified in a grid cell only if at least 11 of the 21 GCMs agree in projecting its existence. (TIF) [file pone.0254879.s004.tif]

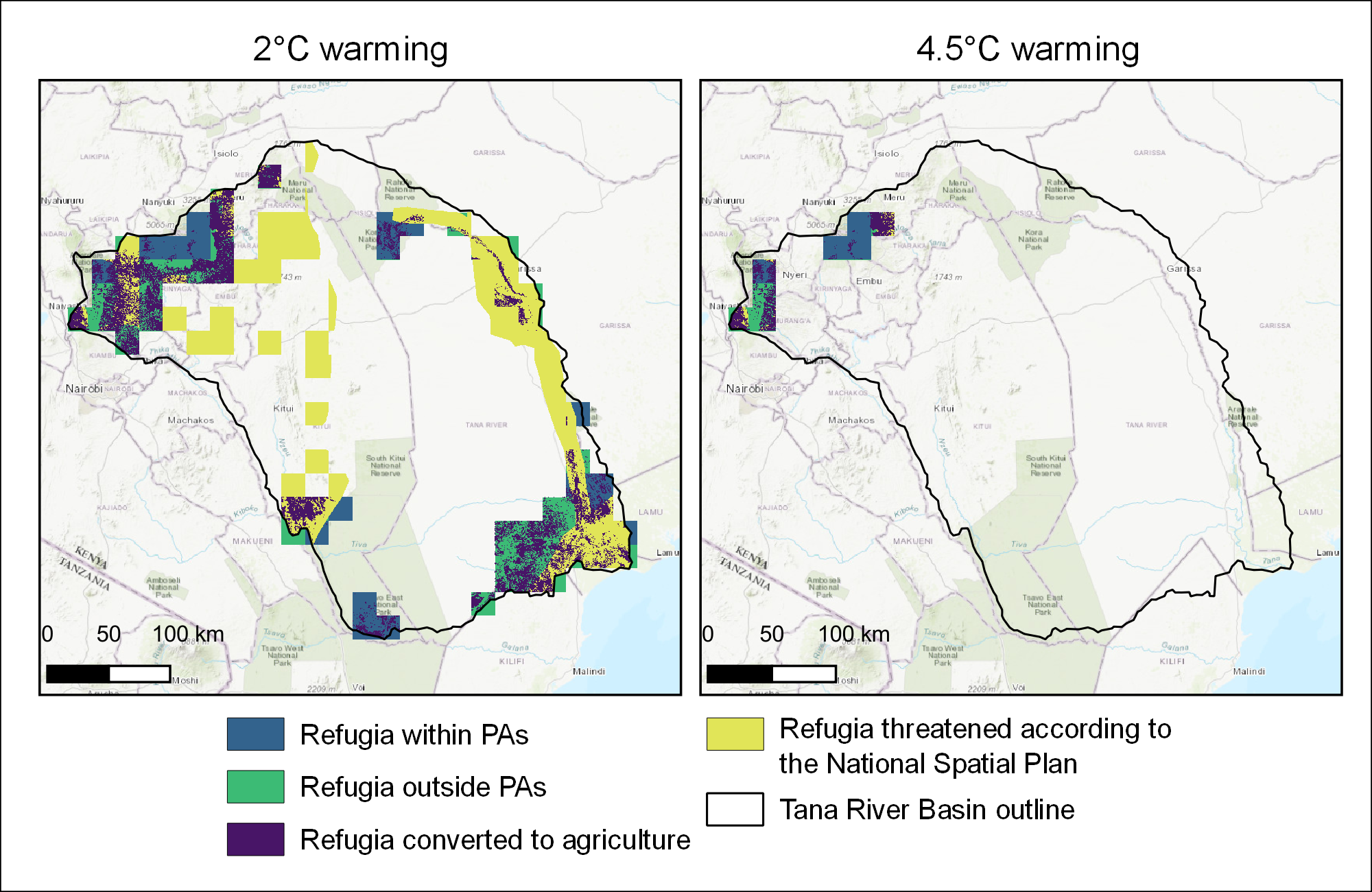

Supplement: S4 Fig — A refugium is identified in a grid cell only if at least 11 of the 21 GCMs agree in projecting its existence. (TIF) [file pone.0254879.s005.tif]

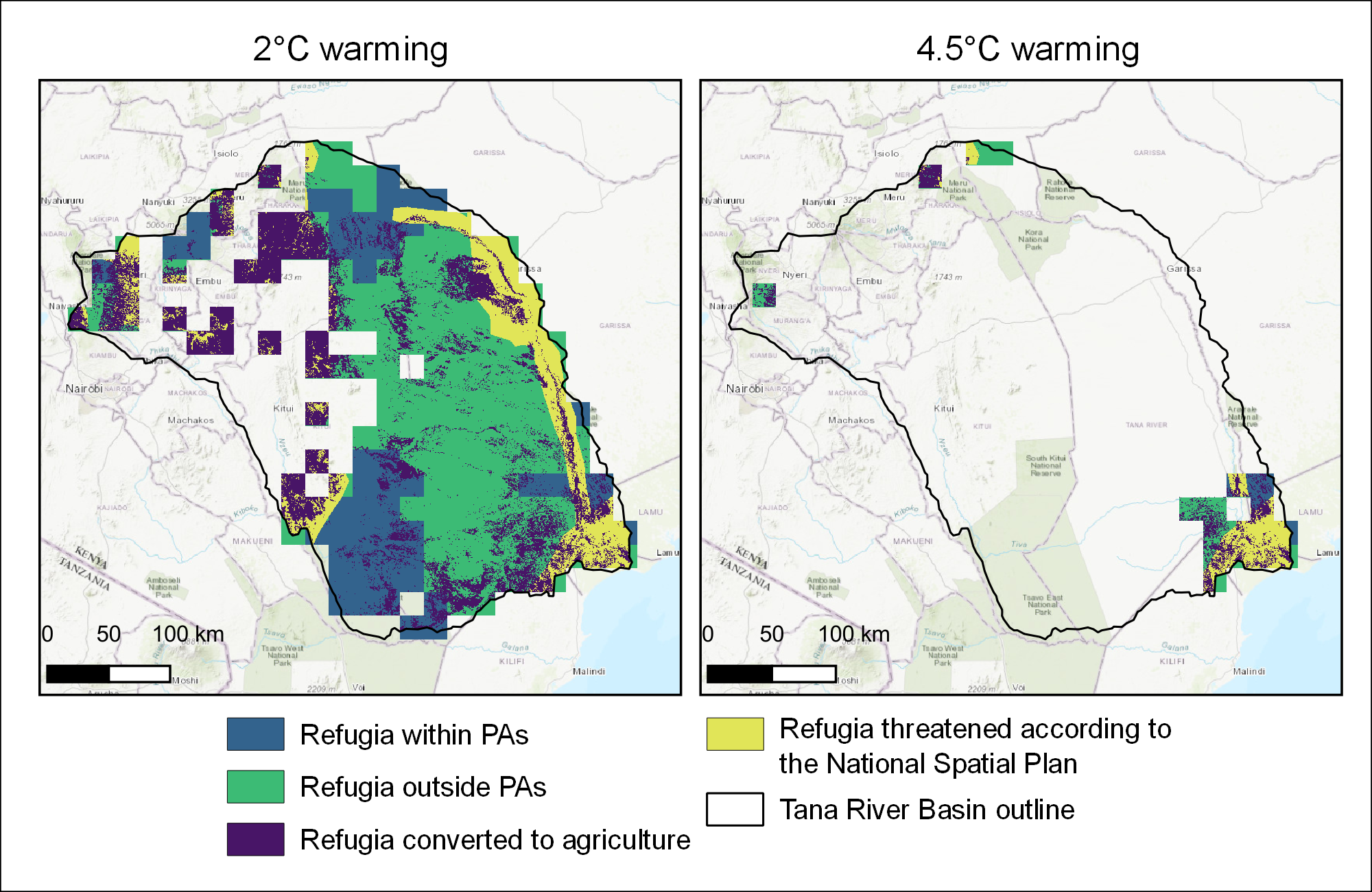

Supplement: S5 Fig — A refugium is identified in a grid cell only if at least 11 of the 21 GCMs agree in projecting its existence. (TIF) [file pone.0254879.s006.tif]

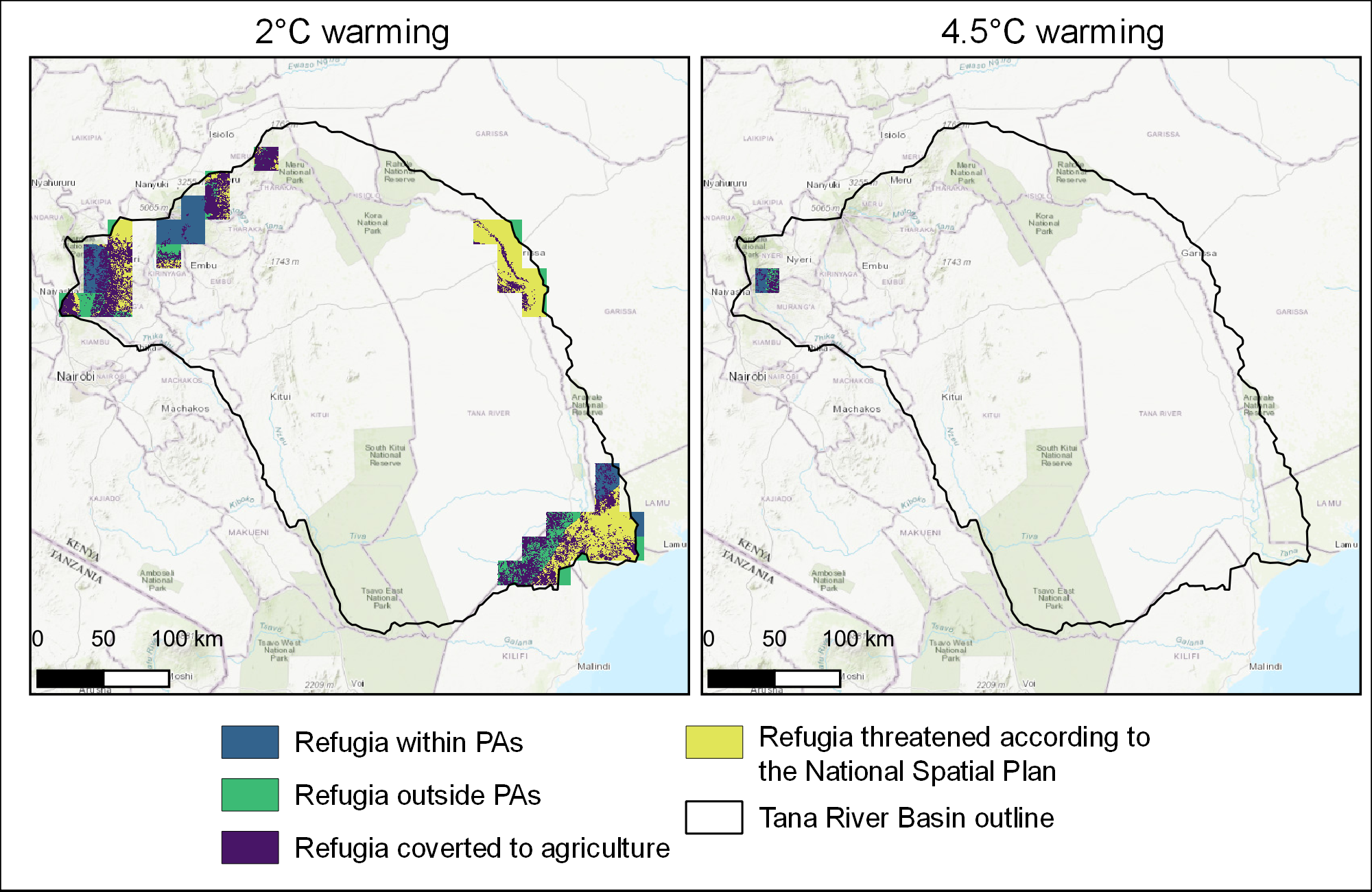

Supplement: S6 Fig — A refugium is identified in a grid cell only if at least 11 of the 21 GCMs agree in projecting its existence. (TIF) [file pone.0254879.s007.tif]

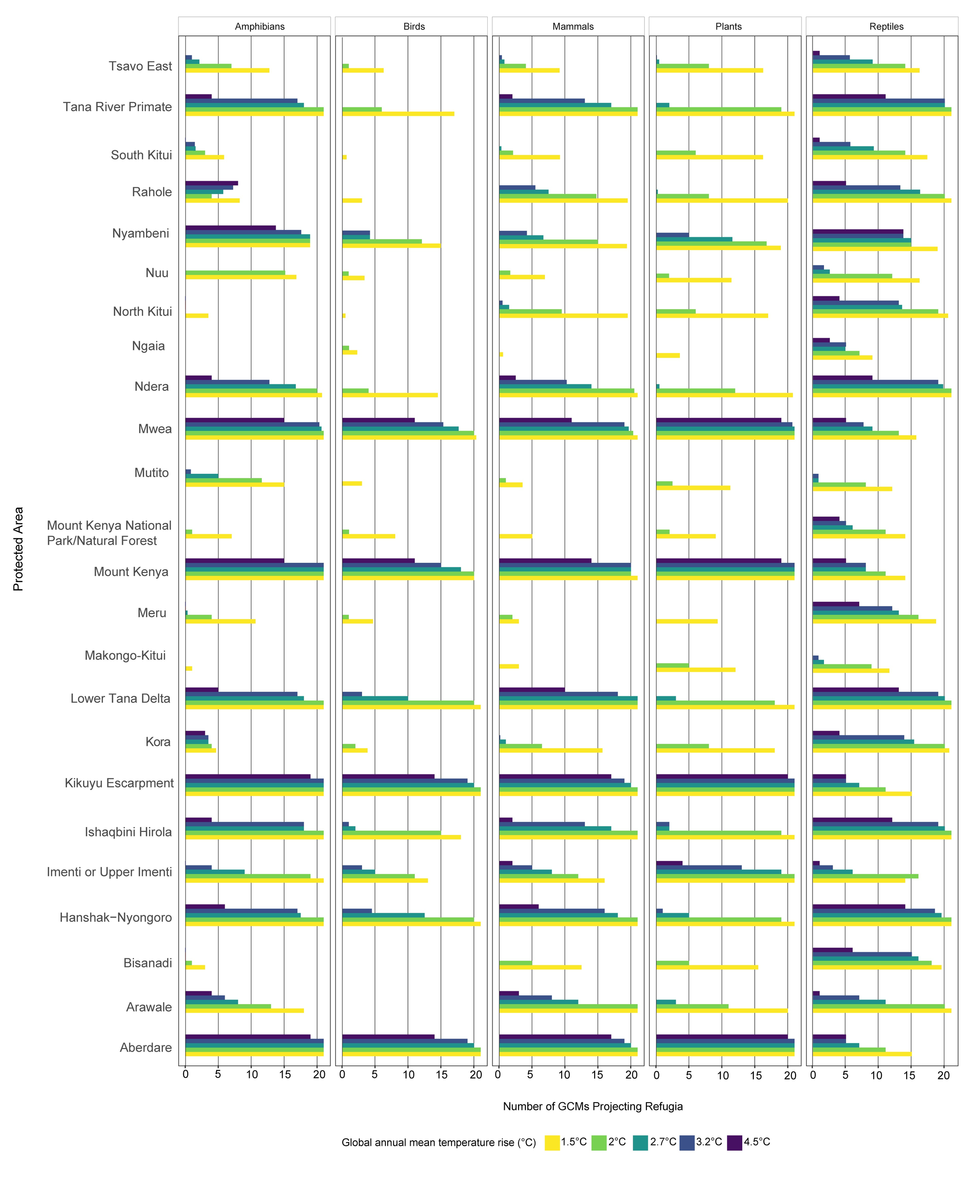

Supplement: S7 Fig — These results are based on a no dispersal scenario. The five classes of species are amphibians, birds, mammals, plants and reptiles. (TIF) [file pone.0254879.s008.tif]

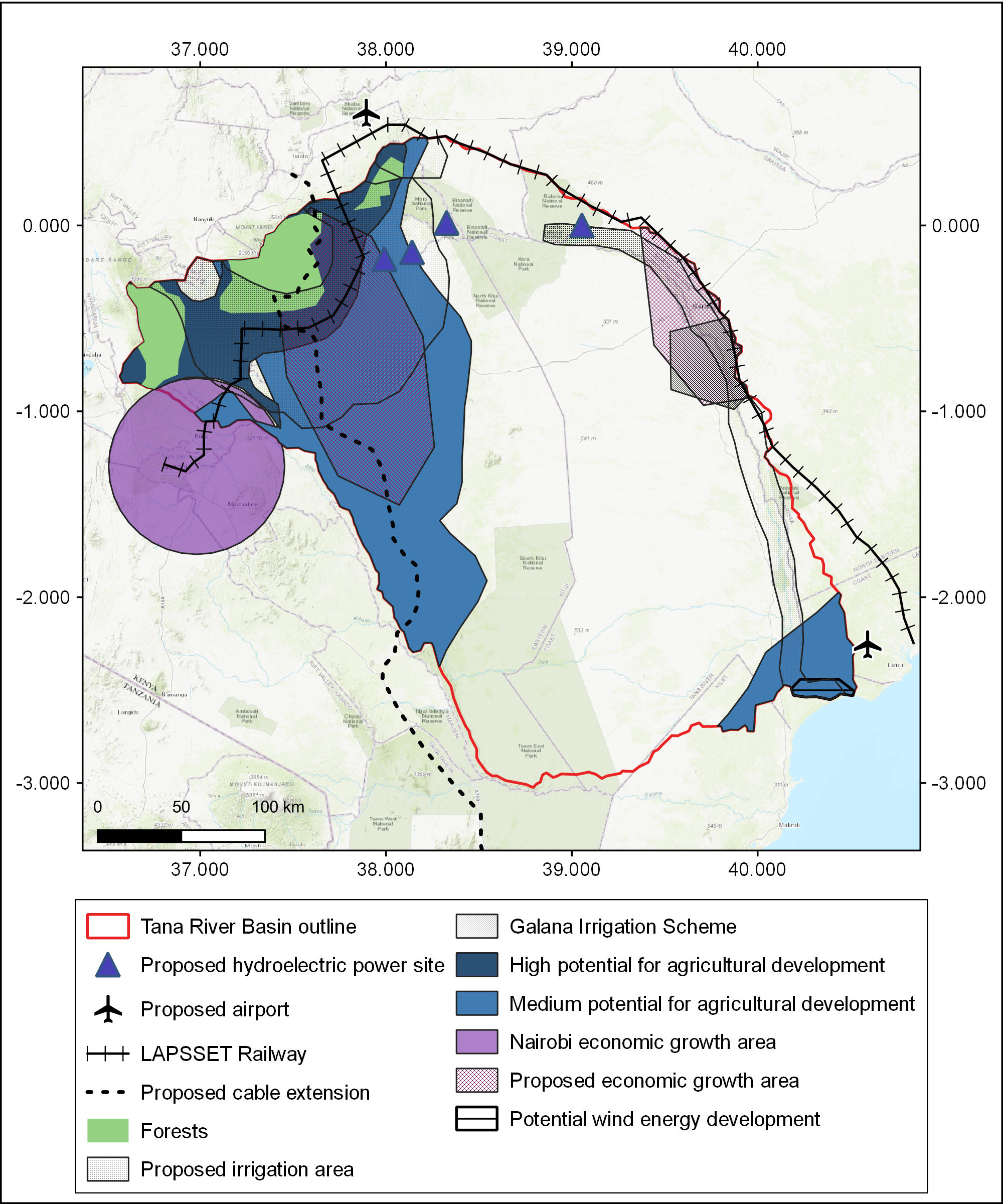

Supplement: S8 Fig — Key features of the National Spatial Plan (Government of Kenya, 2017) within the Tana River Basin boundary were digitised using GIS. (TIF) [file pone.0254879.s009.tif]

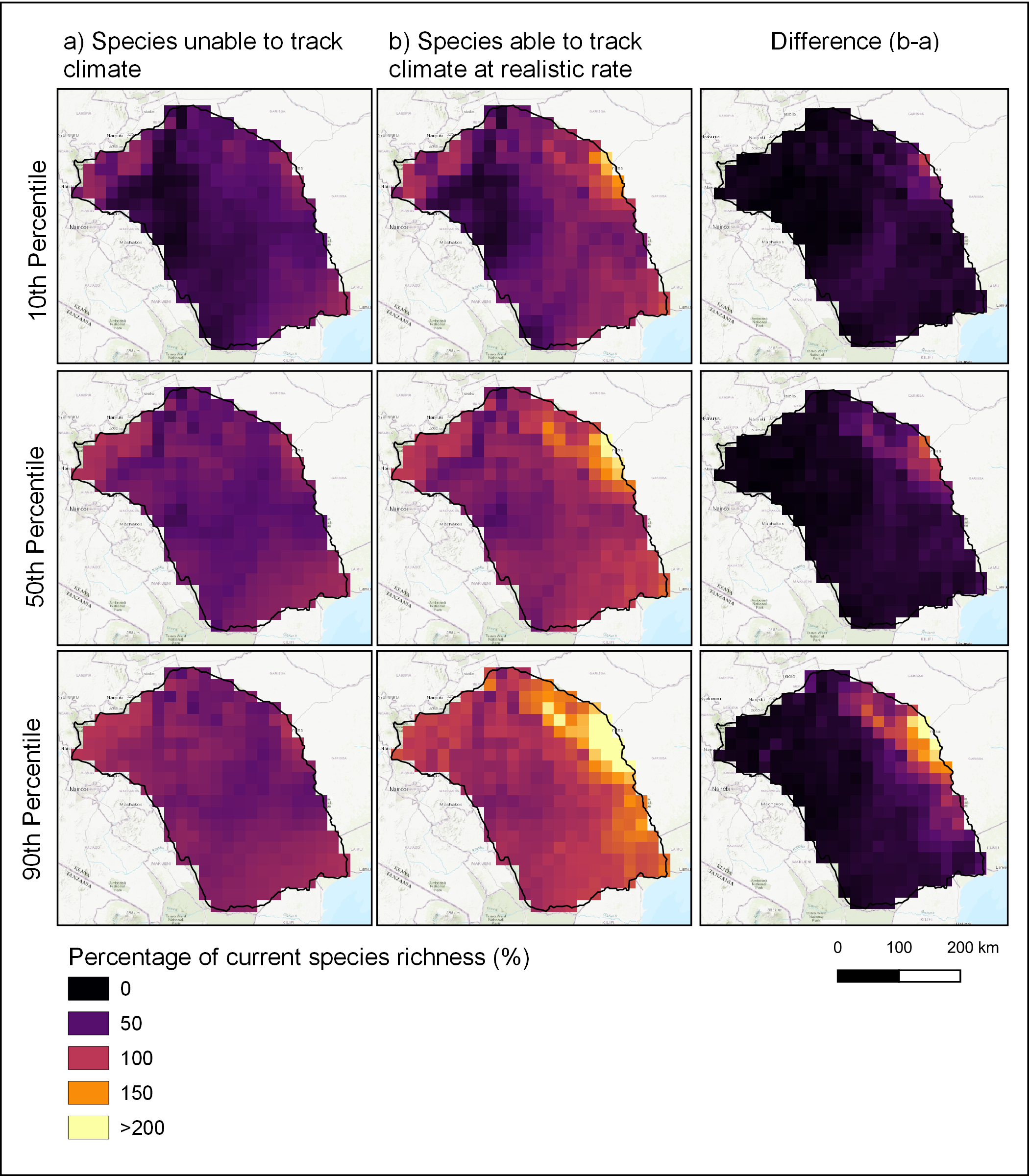

Supplement: S9 Fig — The first column (left) shows species richness remaining when species are not able to shift their ranges, the centre column shows the scenario where species are able to disperse, and the final column (right) shows the difference between the first two. The top panels show the 10th percentile, the middle shows the 50th percentile and the bottom panel shows the 90th percentile. (TIF) [file pone.0254879.s010.tif]

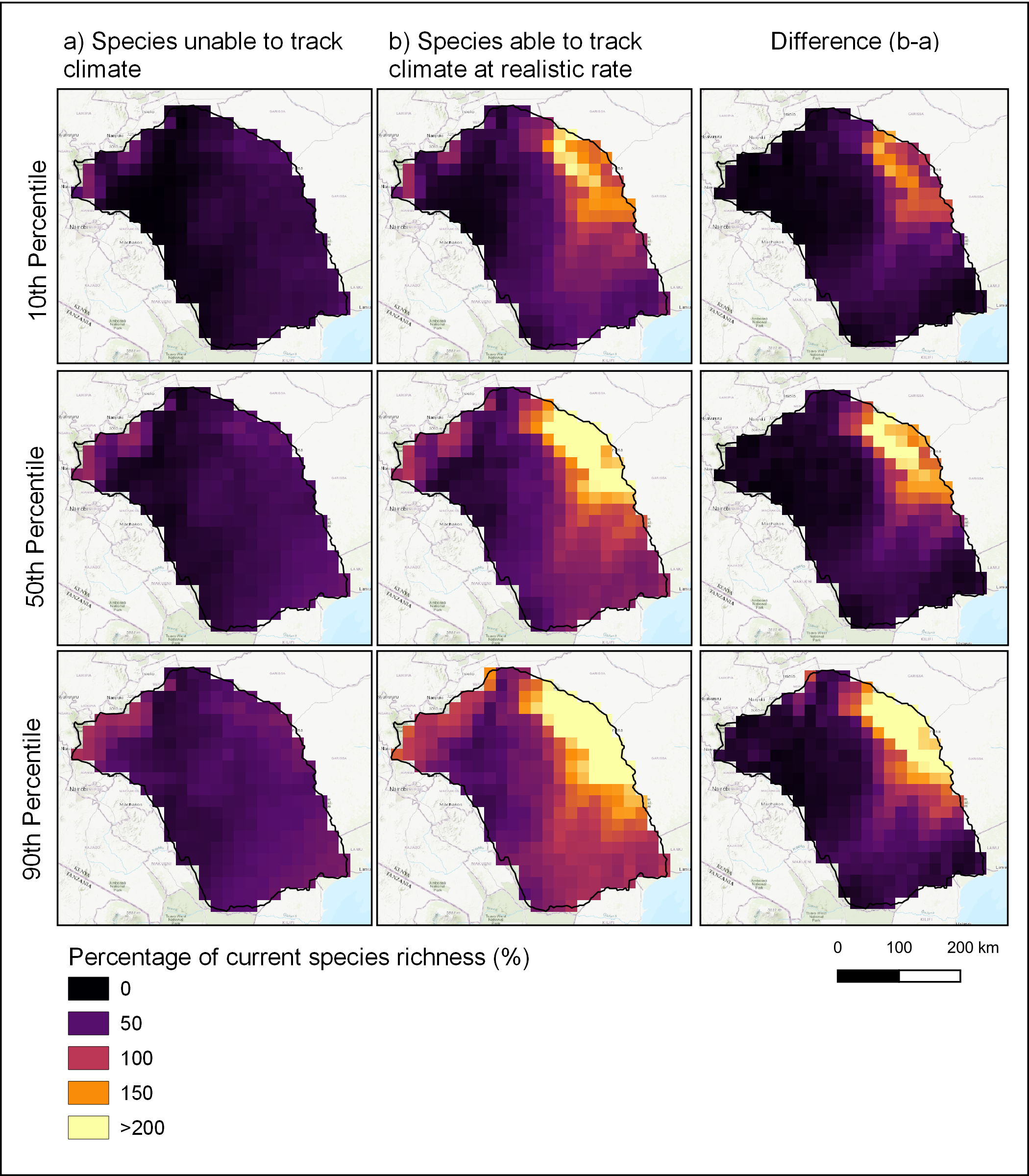

Supplement: S10 Fig — The first column (left) shows species richness remaining when species are not able to shift their ranges, the centre column shows the scenario where species are able to disperse, and the final column (right) shows the difference between the first two. The top panels show the 10th percentile, the middle shows the 50th percentile and the bottom panel shows the 90th percentile. (TIF) [file pone.0254879.s011.tif]

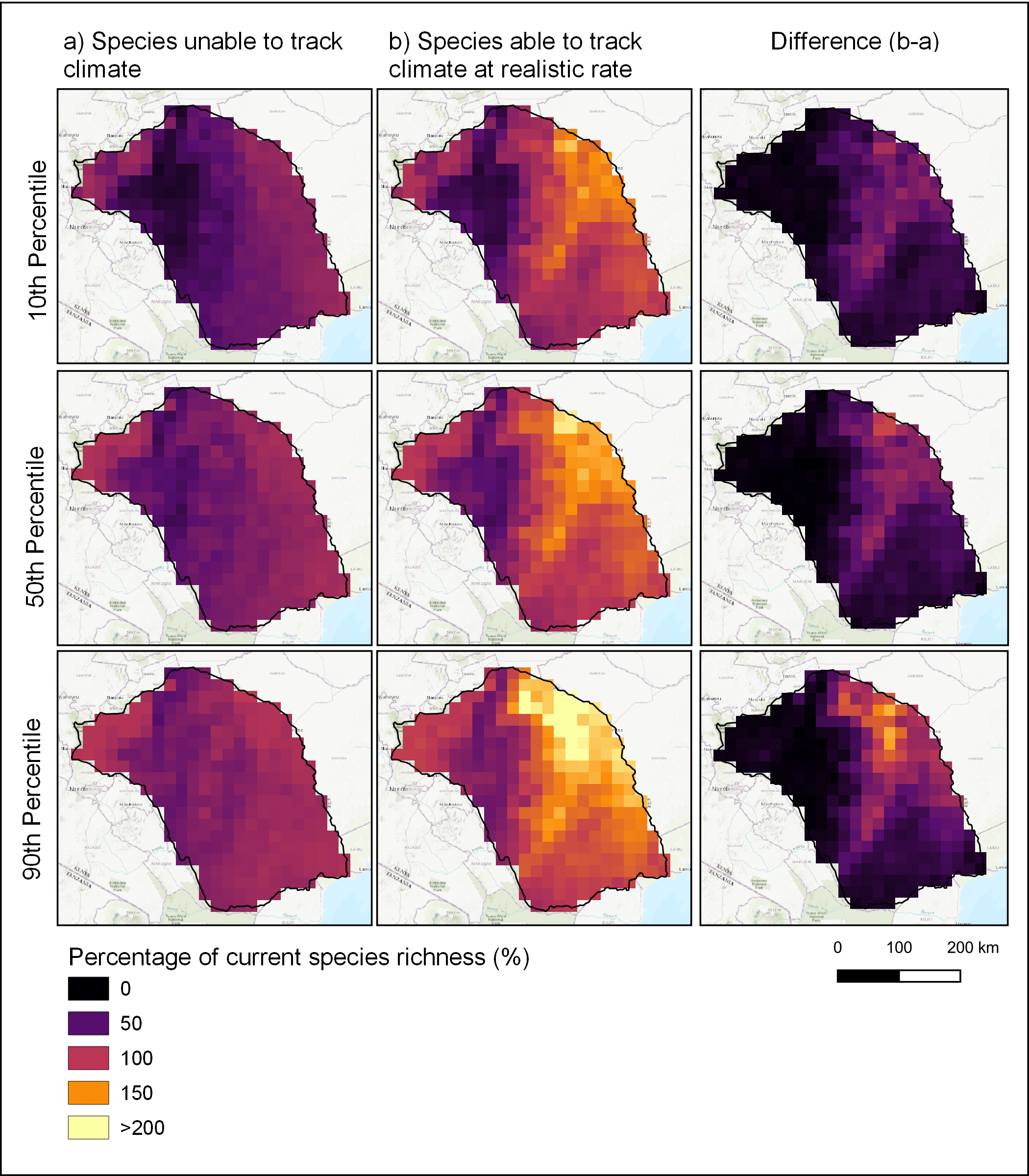

Supplement: S11 Fig — The first column (left) shows species richness remaining when species are not able to shift their ranges, the centre column shows the scenario where species are able to disperse, and the final column (right) shows the difference between the first two. The top panels show the 10th percentile, the middle shows the 50th percentile and the bottom panel shows the 90th percentile. (TIF) [file pone.0254879.s012.tif]

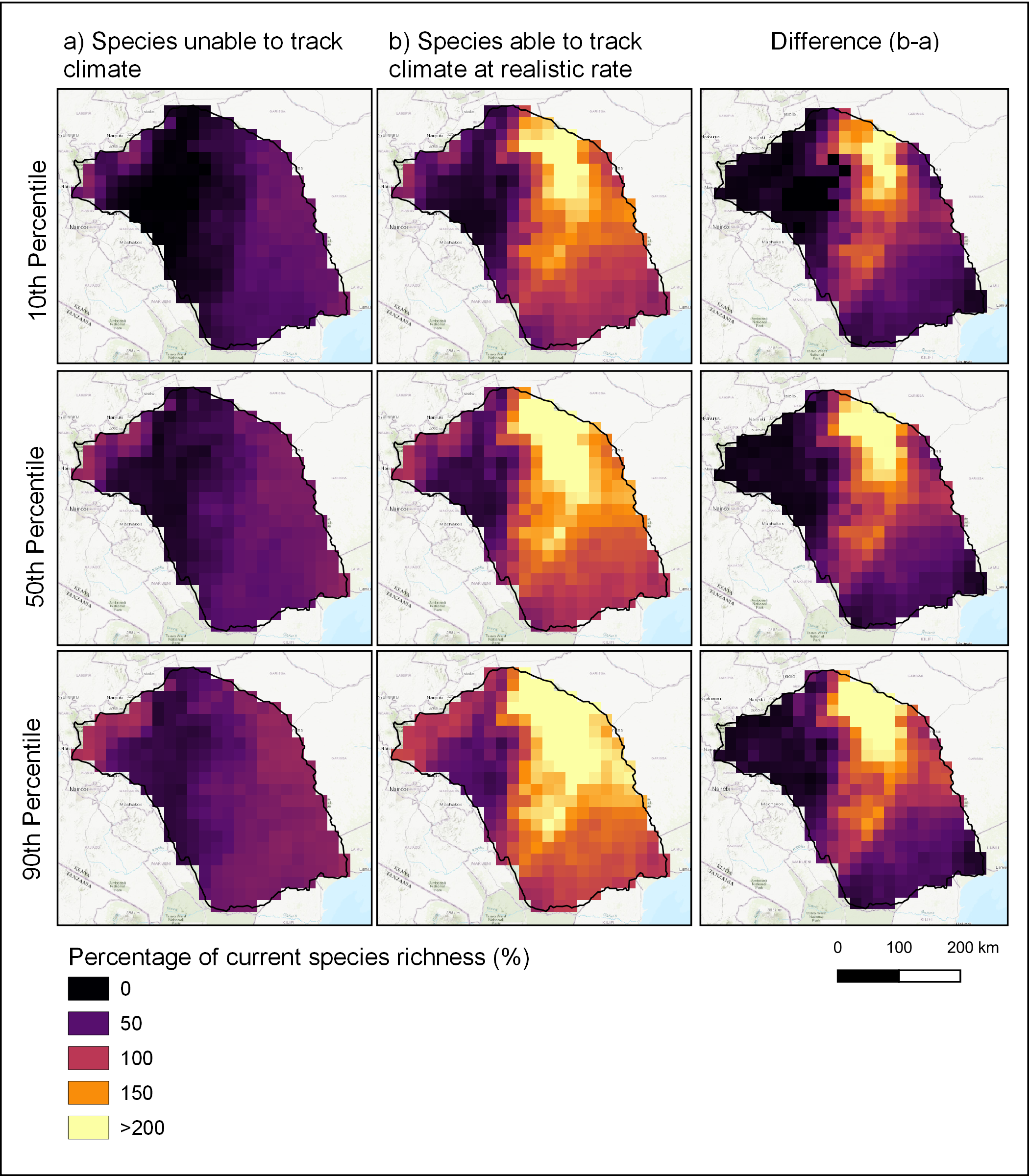

Supplement: S12 Fig — The first column (left) shows species richness remaining when species are not able to shift their ranges, the centre column shows the scenario where species are able to disperse, and the final column (right) shows the difference between the first two. The top panels show the 10th percentile, the middle shows the 50th percentile and the bottom panel shows the 90th percentile. (TIF) [file pone.0254879.s013.tif]
